# Supplementary material for: Comparative multiomics analysis of cell physiological state after culture in a basket bioreactor
Source: Sci Rep. 2022 Nov 23;12:20161. doi: 10.1038/s41598-022-24687-4 (PMC9686226; doi:10.1038/s41598-022-24687-4)

Novogene 诺禾致源

提供领先的基因科技解决方案
Leading Edge Genomic Services & Solutions

蛋白质组学交付数据解读及数据上传概览

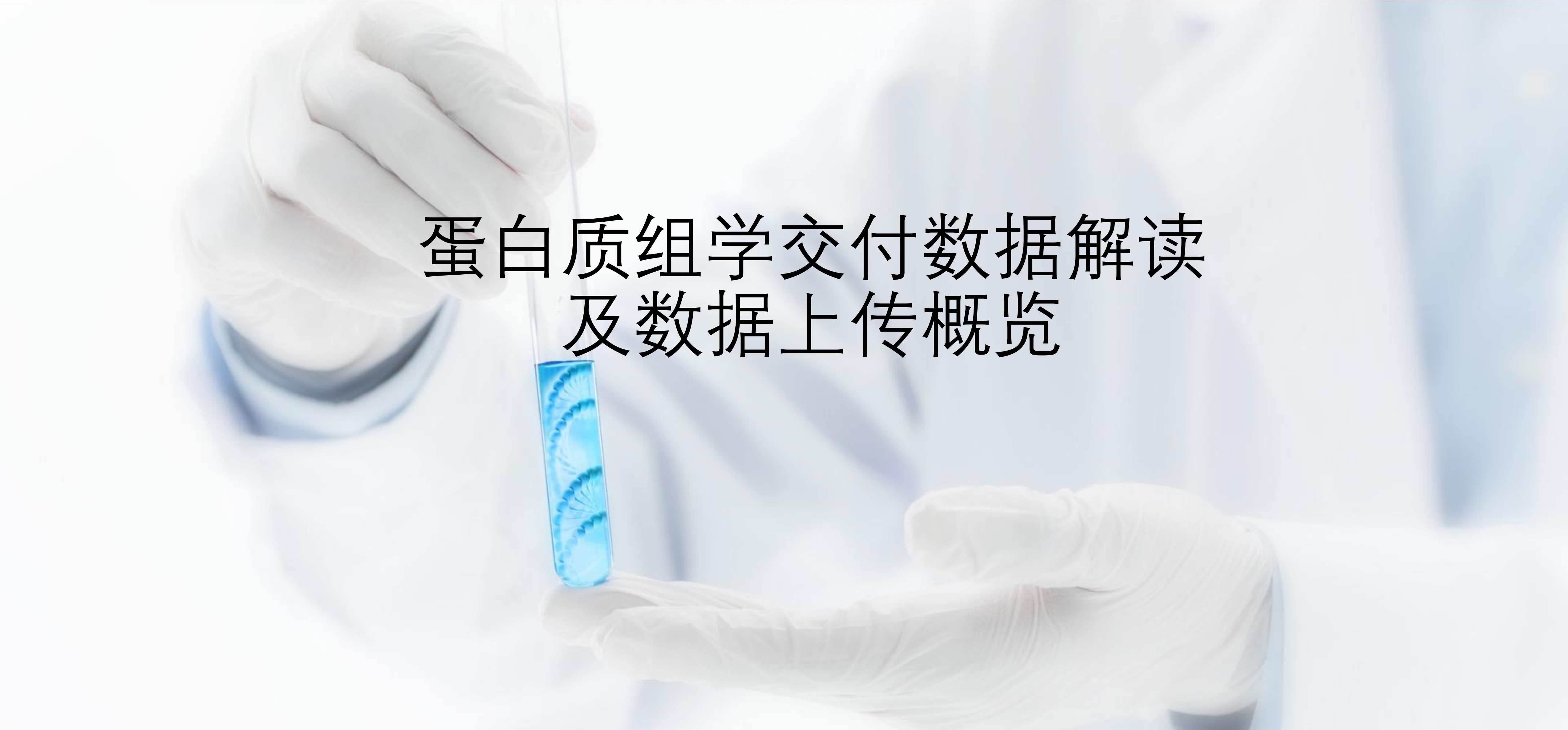

目录

1. 蛋白组学分析内容与技术路线

2. 结题报告解读

3. 结果文件解读

4. 蛋白数据上传

1.分析内容与技术路线（蛋白组学）

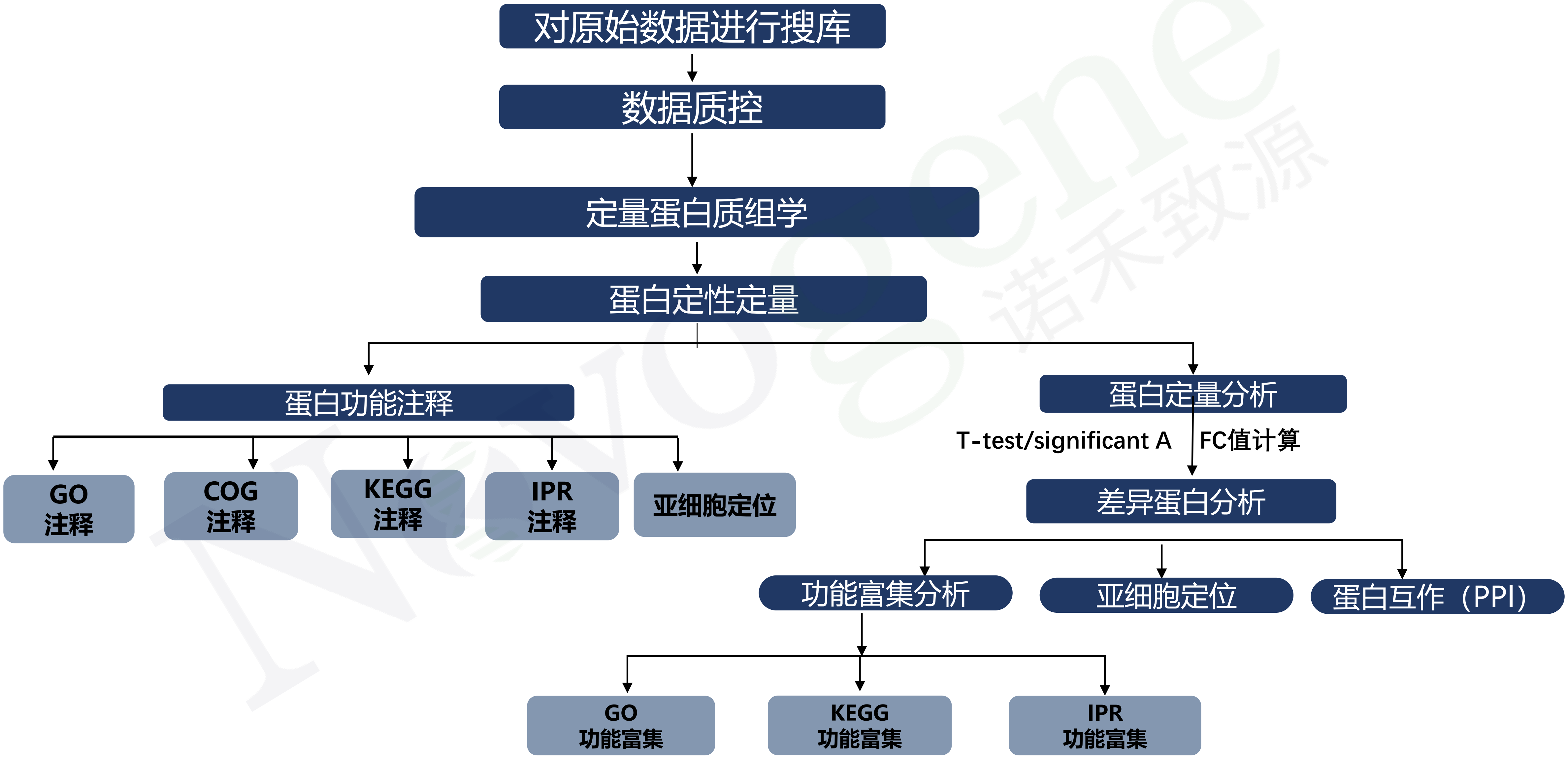

2、结题报告讲解--结题报告和信息分析流程

Novogene 诺禾致源

收起目录 <<

+ 分析概述
+ 工作流程
+ 结果展示及说明
+ 附录
– 参考文献
参考文献

+ 分析概述
– 工作流程
实验流程与分析流程
– 结果展示及说明
数据质控
蛋白功能注释
蛋白定量分析
蛋白差异分析
GO富集分析
KEGG差异分析
结构域富集分析
差异蛋白亚细胞定位分析
差异蛋白互作分析
– 附录

TMT定量蛋白质组结题报告

项目信息

项目编号：X101SC2010xxxx-Z01

项目名称：xxxxTMT分析技术服务（委托）合同

报告时间：2021-01-11

报告编号：X101SC2010xxxx-Z01-J001-B1-43

Novogene 诺禾致源　　北京诺禾致源科技股份有限公司

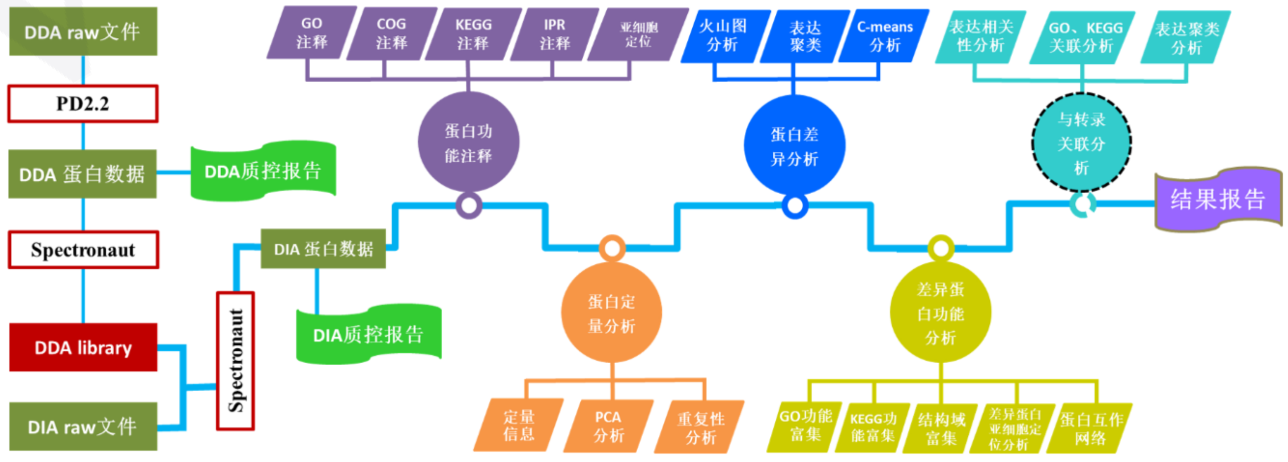

搜库及信息分析流程

2.1 搜库参数和数据质控

蛋白数据库和搜库参数展示

本次使用的数据库：Mus_musculus_uniprot_2020_7_2.fasta(86555 sequences)。

本次使用的搜库软件：Proteome Discoverer2.4。

质谱下机数据格式为*raw，存放质谱数据完整的扫描信息，下机后的raw文件直接导入到Proteome Discoverer软件进行数据库检索，谱肽、蛋白定量。表1为Proteome Discoverer的分析参数：

表1 Proteome Discoverer分析参数

Item	Value
Type of Quantification	Reporter Quantification(TMT)
Enzyme	Trypsin
Max.Missed Cleavage Sites	2
Precursor Mass Tolerance	10 ppm
Fragment Mass Tolerance	0.02 Da
Dynamic Modification	Oxidation/+15.995 Da (M) and TMT /+229.163 Da (K,Y)
N-Terminal Modification	Acetyl/+42.011 Da (N-Terminal) and TMT 10plex/+229.163 Da (K, Y) and Met-loss/-131.040Da(M) and Met-loss+Acetyl/-89.030 D[illegible]
Static Modification	Carbamidomethyl/+57.021 Da (C)

质谱下机数据需要进行蛋白数据库搜索。数据库的选择是整个信息分析流程中关键的一步，最终鉴定到的蛋白质序列都来源于被选择的数据库，目前比较常用的数据库包括：NCBI 、UniProt 数据库等。

数据质控

在质谱数据检索后，要进行搜库结果的检验，从而考察数据的质量。检验的内容：

- 肽段信息；
- 蛋白信息

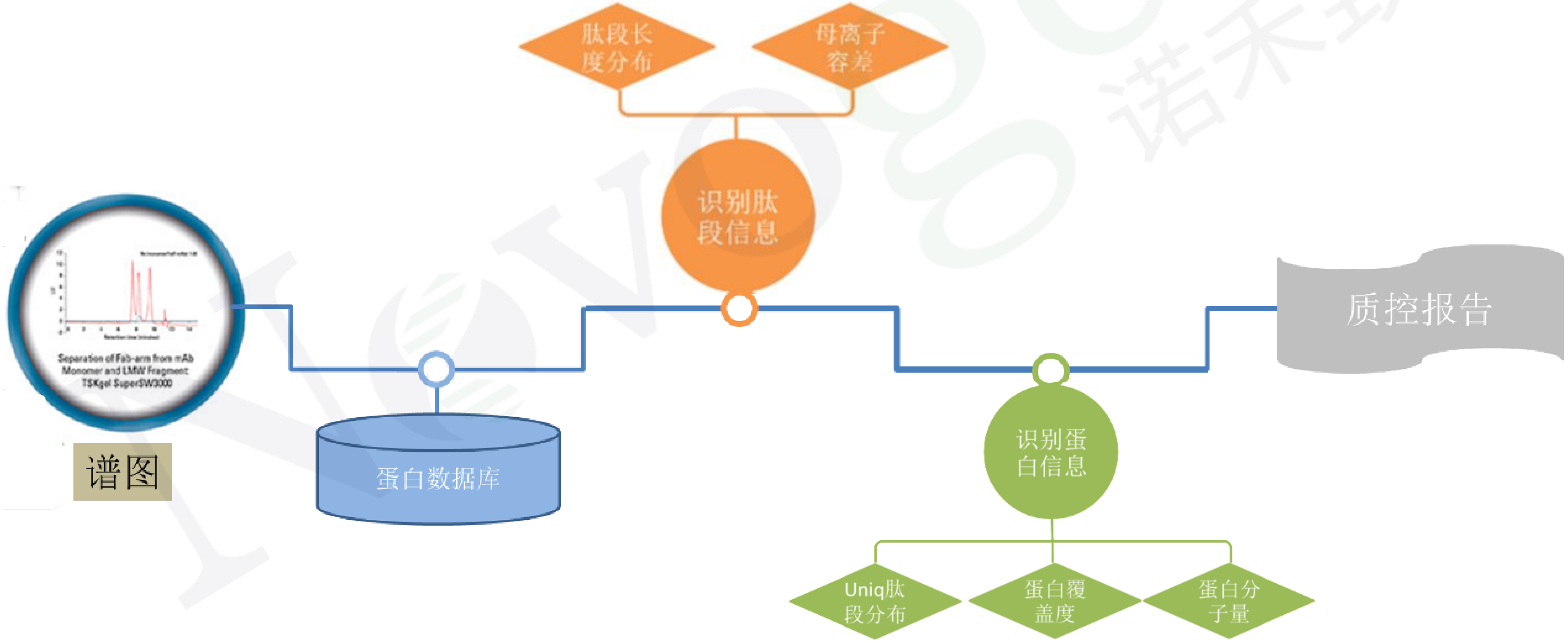

肽段信息

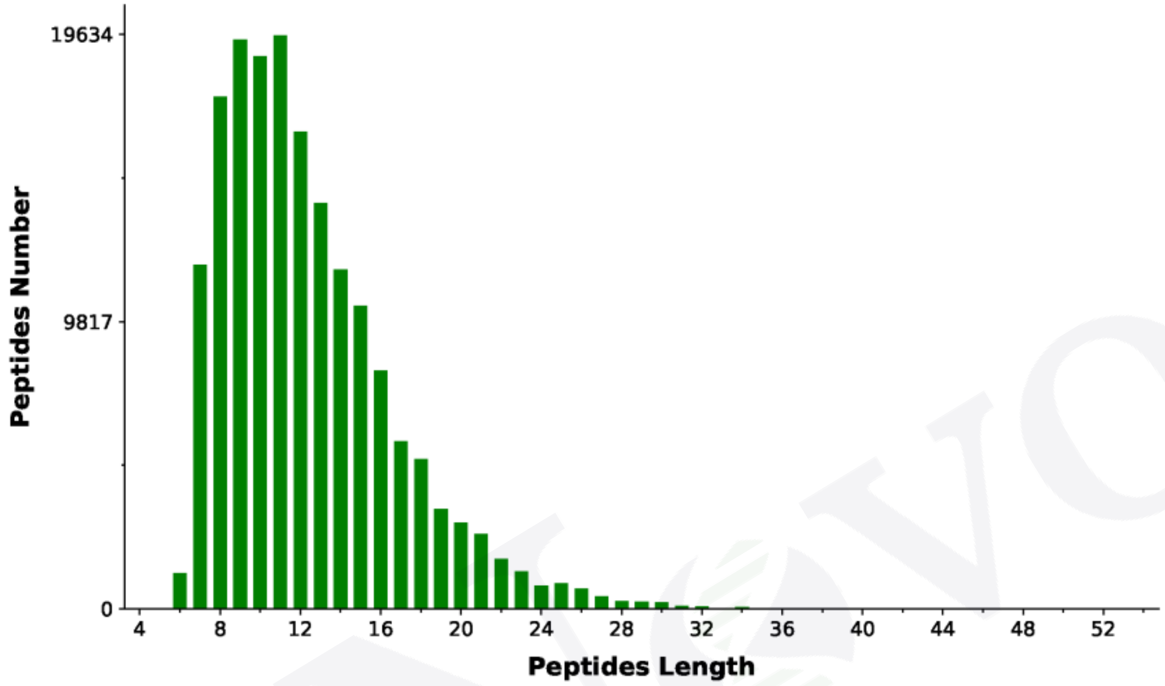

图 a 肽段长度范围分布图

横坐标为肽段氨基酸残基数，纵坐标为该长度肽段的个数。肽段长度主要分布在7-25之间。

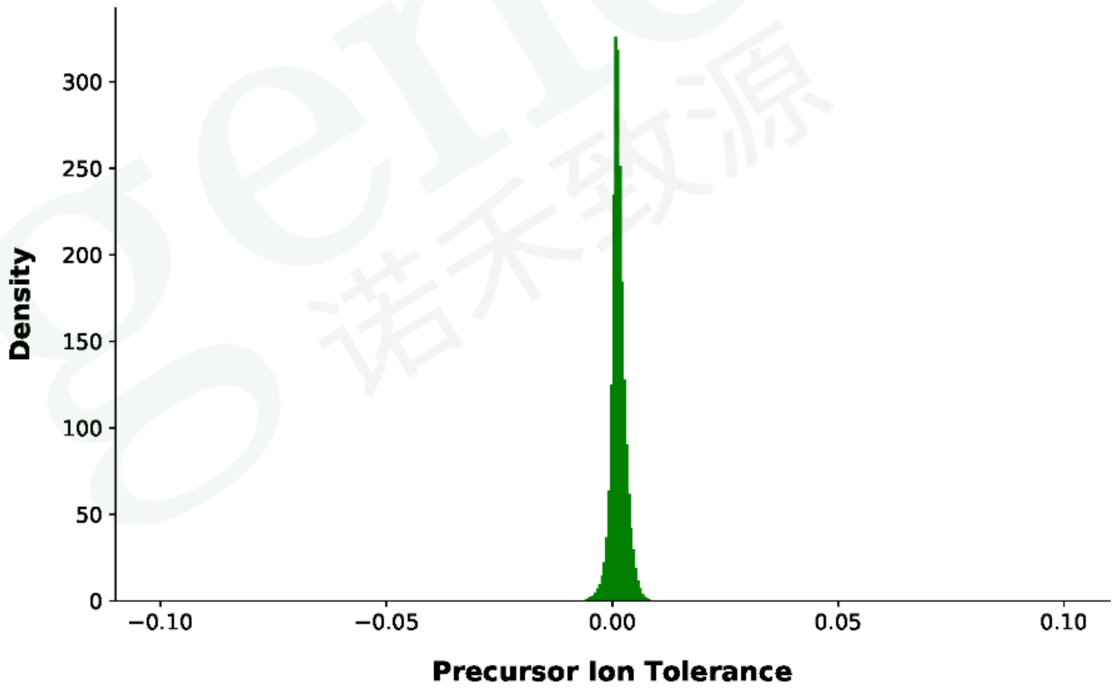

图 b 母离子质量容差分布

横坐标为质量偏差，纵坐标为相应误差的母离子密度分布。峰型越集中在0附近，说明质量偏差越小。

➢ 横坐标是含有Unique肽段的个数，纵坐标是随着Unique肽段个数增多时，含有Unique肽段的蛋白占总蛋白的累积占比。

➢ 所以当曲线增加越缓，说明Unique 肽段数量越多，鉴定到的可靠蛋白更多

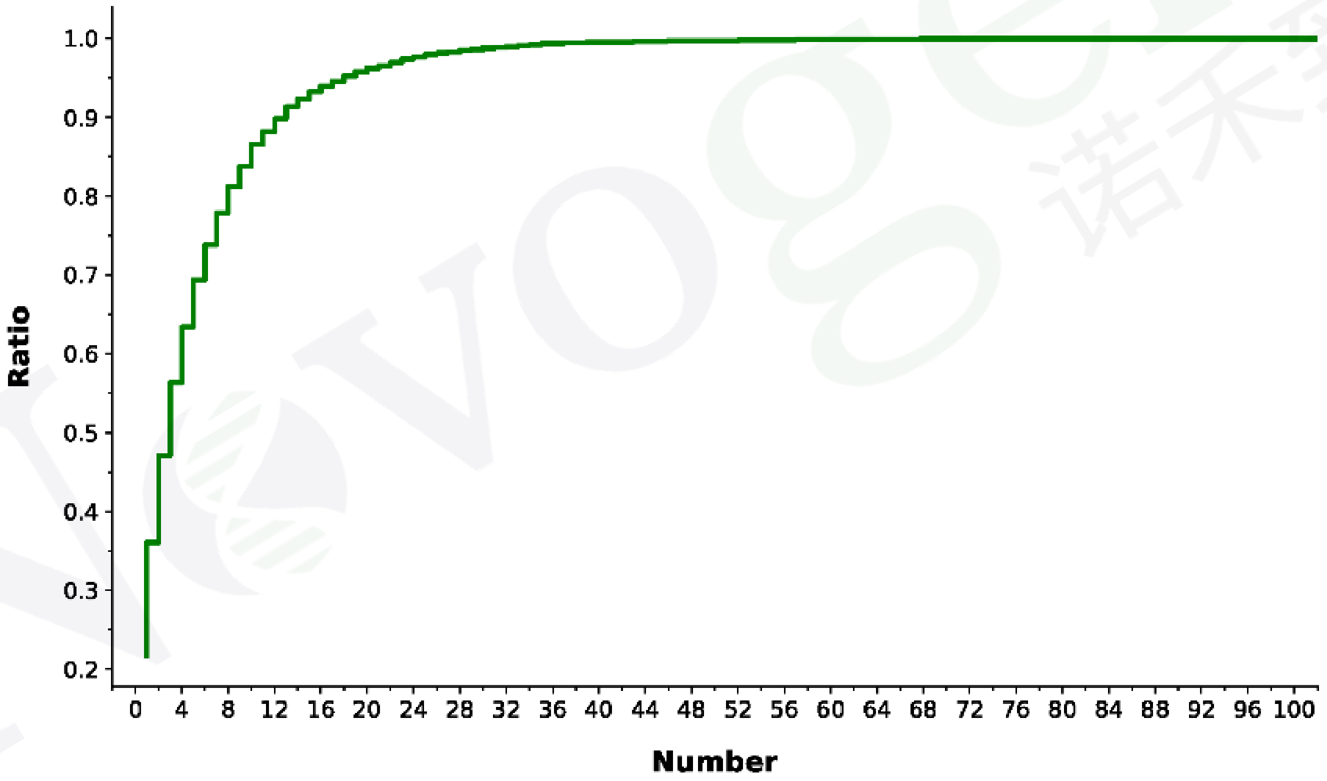

图 e 鉴定蛋白中的unique肽段数分布图

蛋白信息

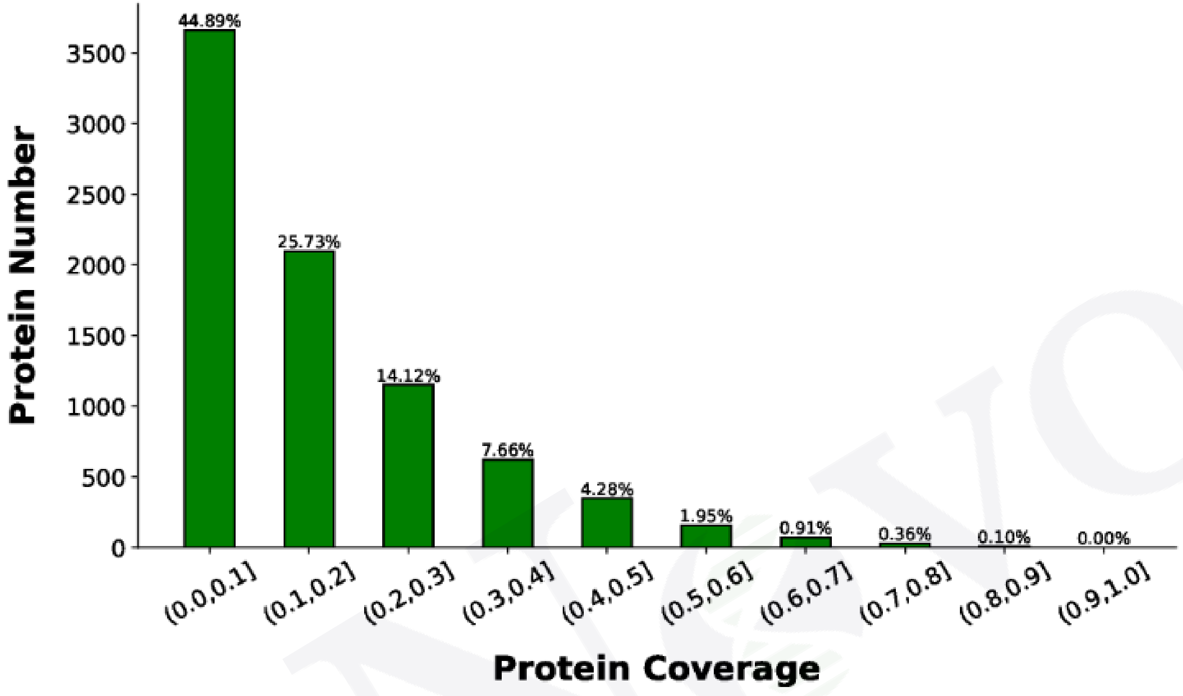

图 c 蛋白覆盖度分布

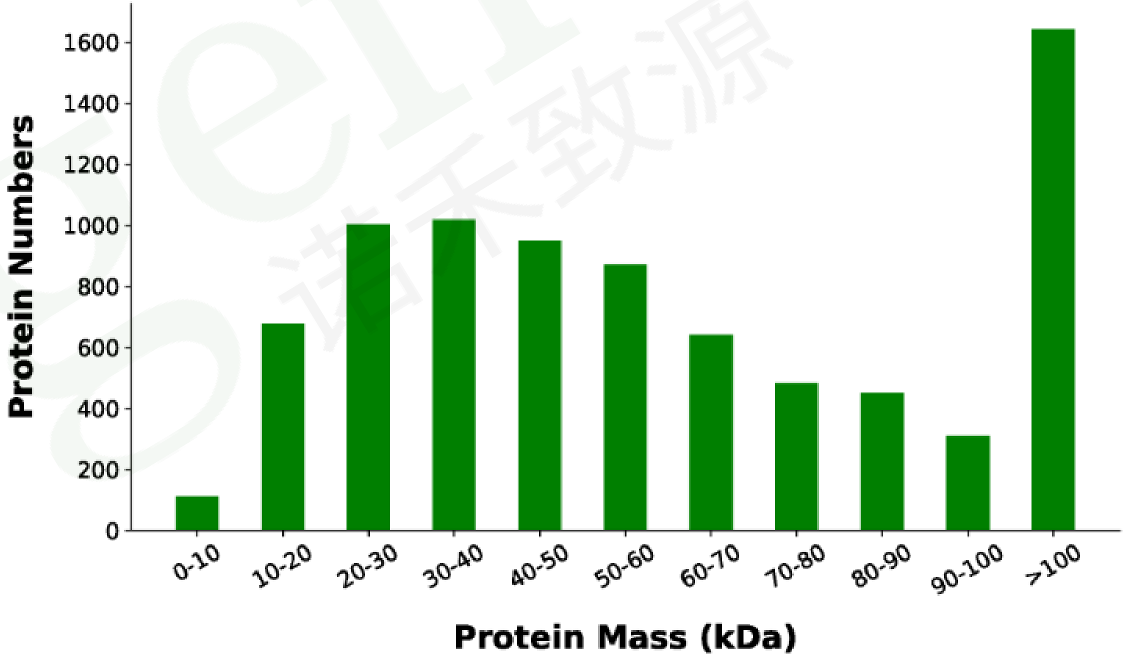

图 d 蛋白分子量分布

横坐标为蛋白覆盖度的区间（检测到的肽段所覆盖的该蛋白长度 / 该蛋白的全长），纵坐标为相应区间包含的蛋白数目。

横坐标为鉴定到的蛋白质分子质量（单位: 千道尔顿，kDa），纵坐标为鉴定到的蛋白质数量。

数据质控

- 0.raw.data
- 1.data_control
- 2.func.anno
- 3.ProtExprQuantification
- 4.DiffExprAnalysis
- 5.GOenrichment
- 6.KEGGenrichment
- 7.IPRenrichment
- 8.Subcellular_Diff
- 9.PPI
- results_dir
- results_readme

```
1.data_control/
├── 1.data_control_README.txt
├── cov_prot.pdf
├── cov_prot.png
├── dmass_distri.pdf
├── dmass_distri.png
├── len_prot.pdf
├── len_prot.png
├── MW_distri.pdf
├── MW_distri.png
├── quality.xls
├── run1_usage_data.txt
├── uniq_distri.pdf
└── uniq_distri.png
```

Sequence	Delta_Da	MW_kDa	uniq	coverage
[R].iYEIE	0.00136	141.9	93	67
[R].aGATV	0.00064	77.5	50	70
[R].aGATV	0.0008	56.8	43	84
[R].aGATV	0.00081	128.1	61	55
[K].rSETF	-0.00113	66.5	50	71
[R].gDITE	0.00273	104	54	57

Sequence：所有谱肽的序列
Delta_Da：所有一级质谱母离子的误差
MW_kDa：有鉴定蛋白的分子量
Uniq: 所有鉴定蛋白所含的Unique肽段数
Coverage：所有鉴定蛋白的覆盖度

2.2 蛋白定性和功能注释

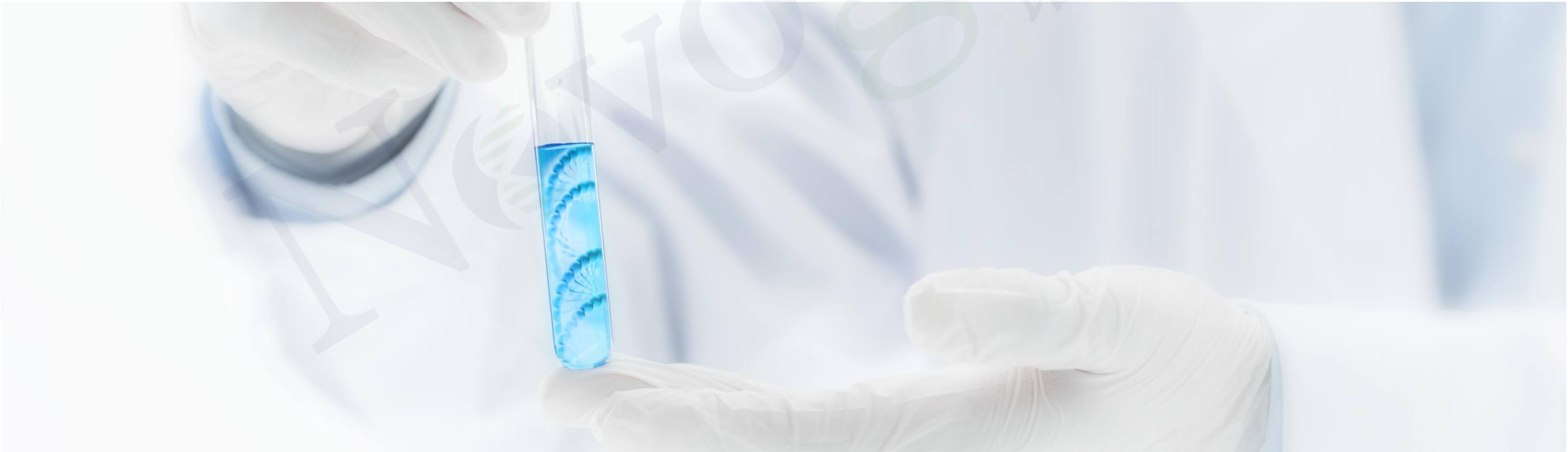

蛋白定性

蛋白鉴定总览

定性项目

Name	Total spectra	Matched spectrum	Peptide	Identified protein
OE_70k	17594	467	368	132
OE_40k	19852	977	895	321

标记定量项目

Run name	Total spectra	Matched spectrum	Peptide	Identified protein
run1	404496	93183	33012	3962
run2	400949	96012	33437	3887
ALL	-	-	-	4481

非标记类定量项目: Label-free DIA

Name	Total spectra	Matched spectrum	Peptide	Identified protein
ALL	340695	80787	40629	7798

Name	Peptide	Identified protein
ALL	36070	5998

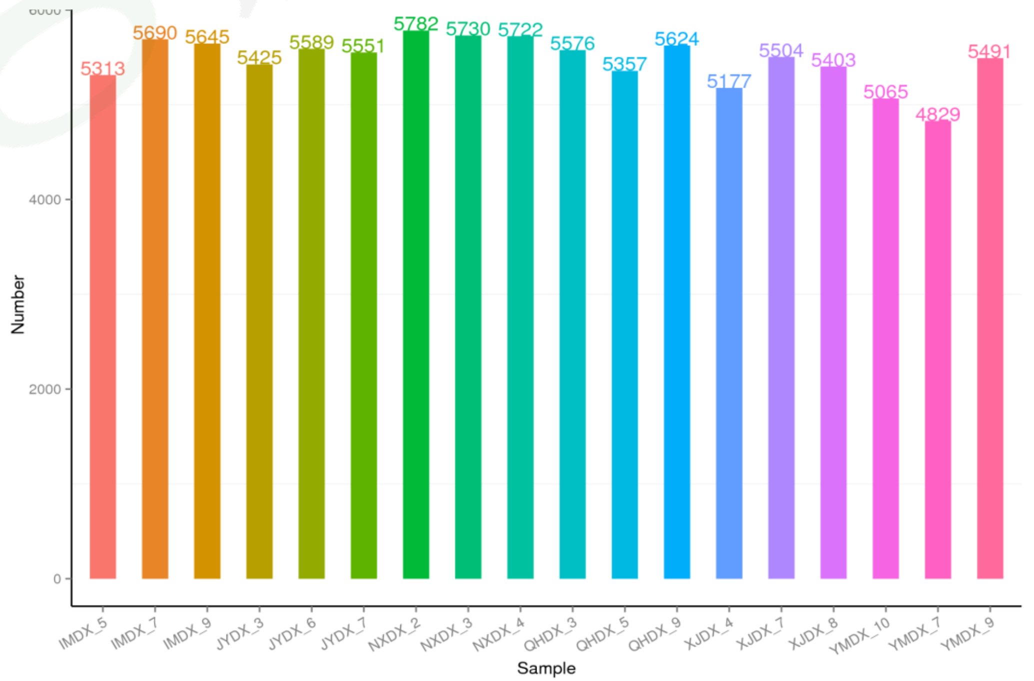

注：Run name: 分run信息；Name：样品；Total Spectra：二级谱图总数；Matched spectrum：有效谱图数；Peptide：鉴定到的肽段的数量；Identified Protein：鉴定到的蛋白质数量。

蛋白功能注释

目前注释的通用功能数据库主要有GO、KEGG、COG等。通过利用这些数据库对鉴定到的蛋白质进行功能注释，以了解不同蛋白质的功能特性。

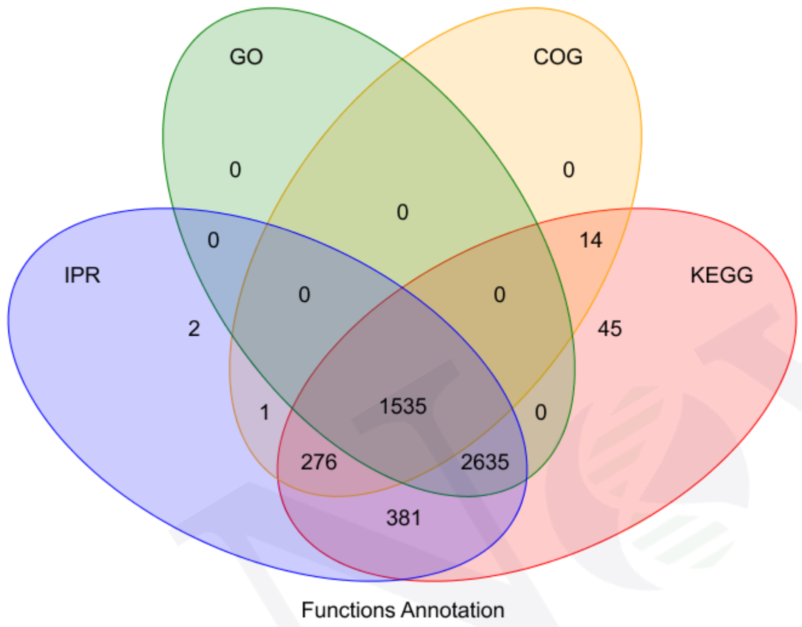

图中每个圈代表一个数据库的注释结果，重叠部分代表多个数据库共同注释到的蛋白，非重叠部分代表相应数据库独有注释到的蛋白

注释图片展示和对应的结果目录

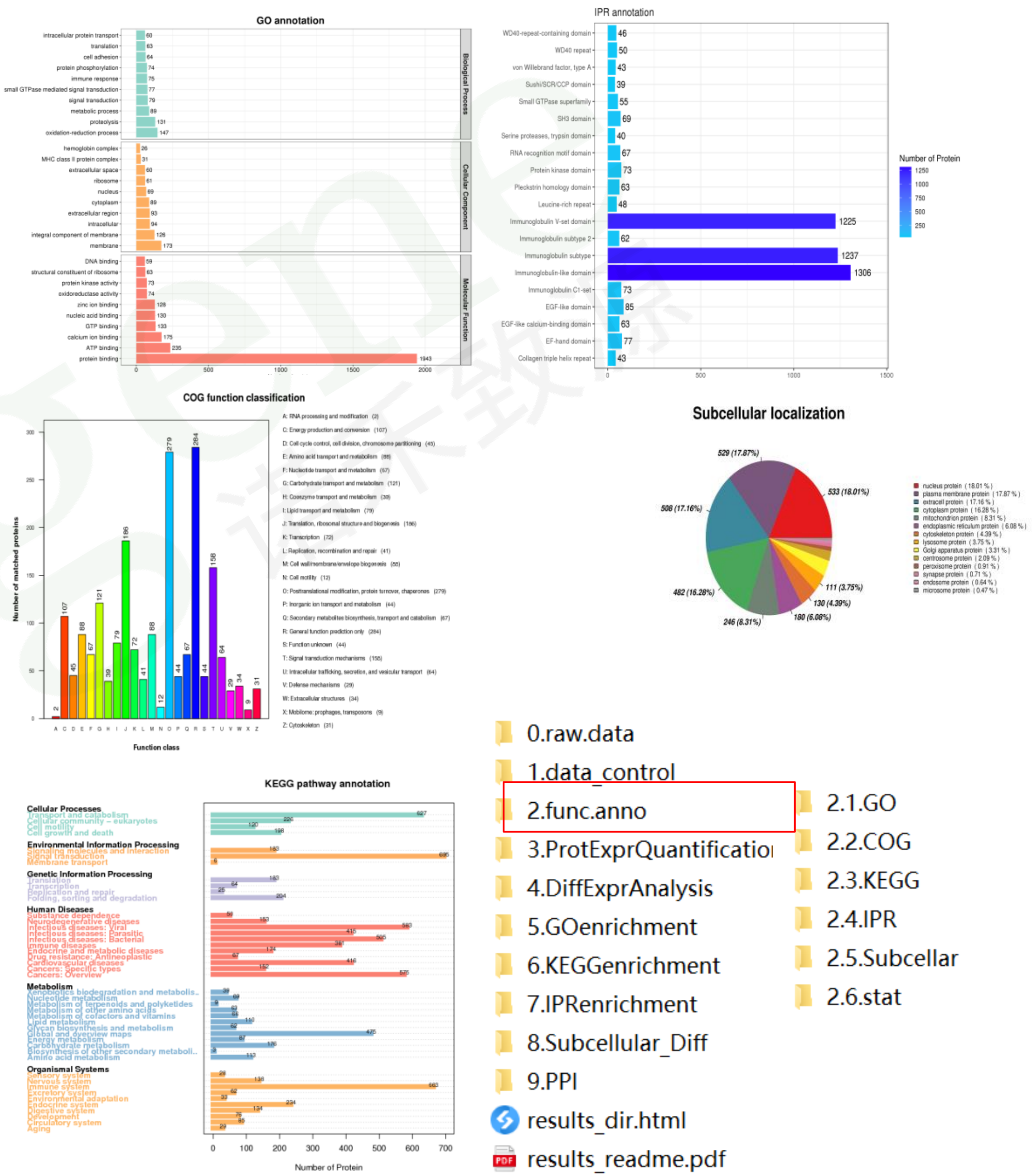

GO注释

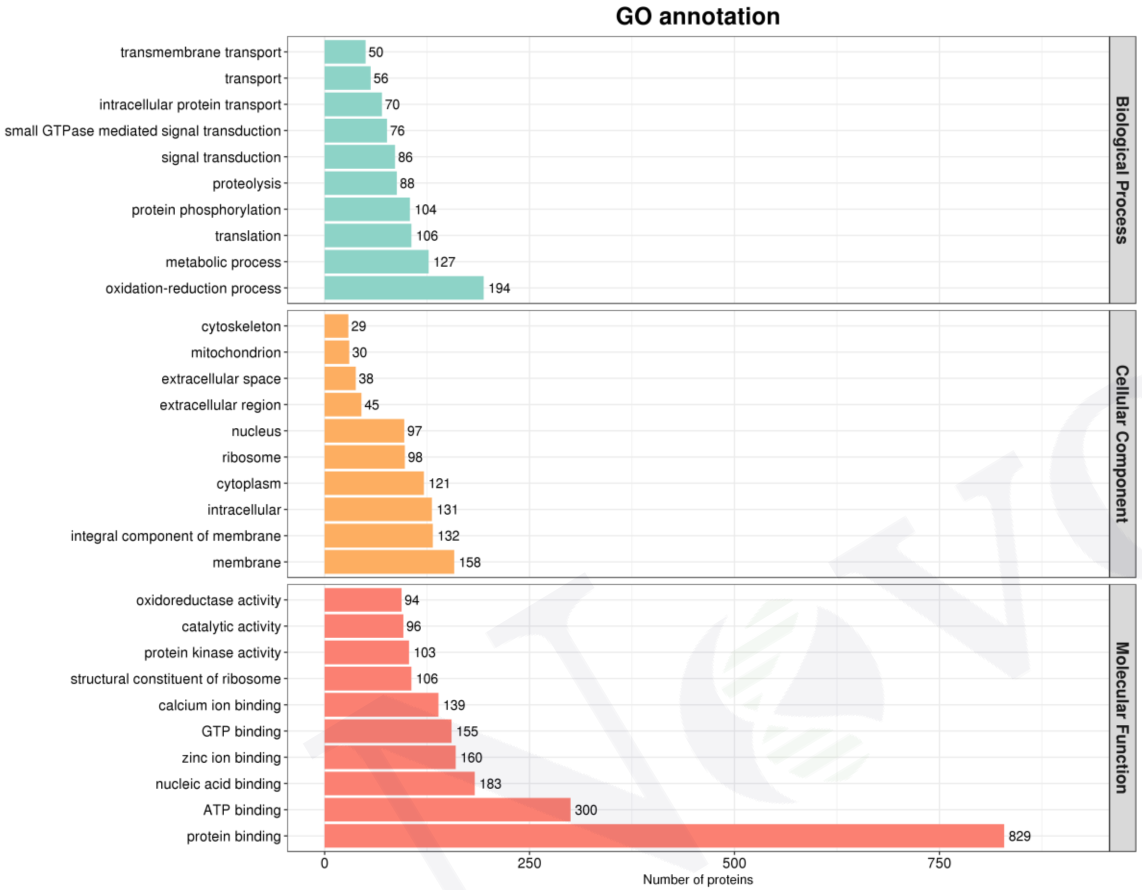

GO（ GeneOntology ）分为三大类：

- 细胞组分（Cellular Component）：用于描述亚细胞结构、位置和大分子复合物，如核仁、端粒和识别起始的复合物；
- 分子功能（Molecular Function）：用于描述基因、基因产物个体的功能，如与碳水化合物结合或 ATP 水解酶活性等；
- 生物过程（Biological Process）：用来描述基因编码的产物所参与的生物过程，如有丝分裂或嘌呤代谢等。

IPR注释

- Interproscan是蛋白质结构域和功能注释最常用的软件之一，是EBI开发的一个集成了蛋白质家族、结构域和功能位点的非冗余数据库。
- Interproscan整合了一些最常用的结构域数据库，利用模式结构或特征进行功能未知蛋白的结构域注释。

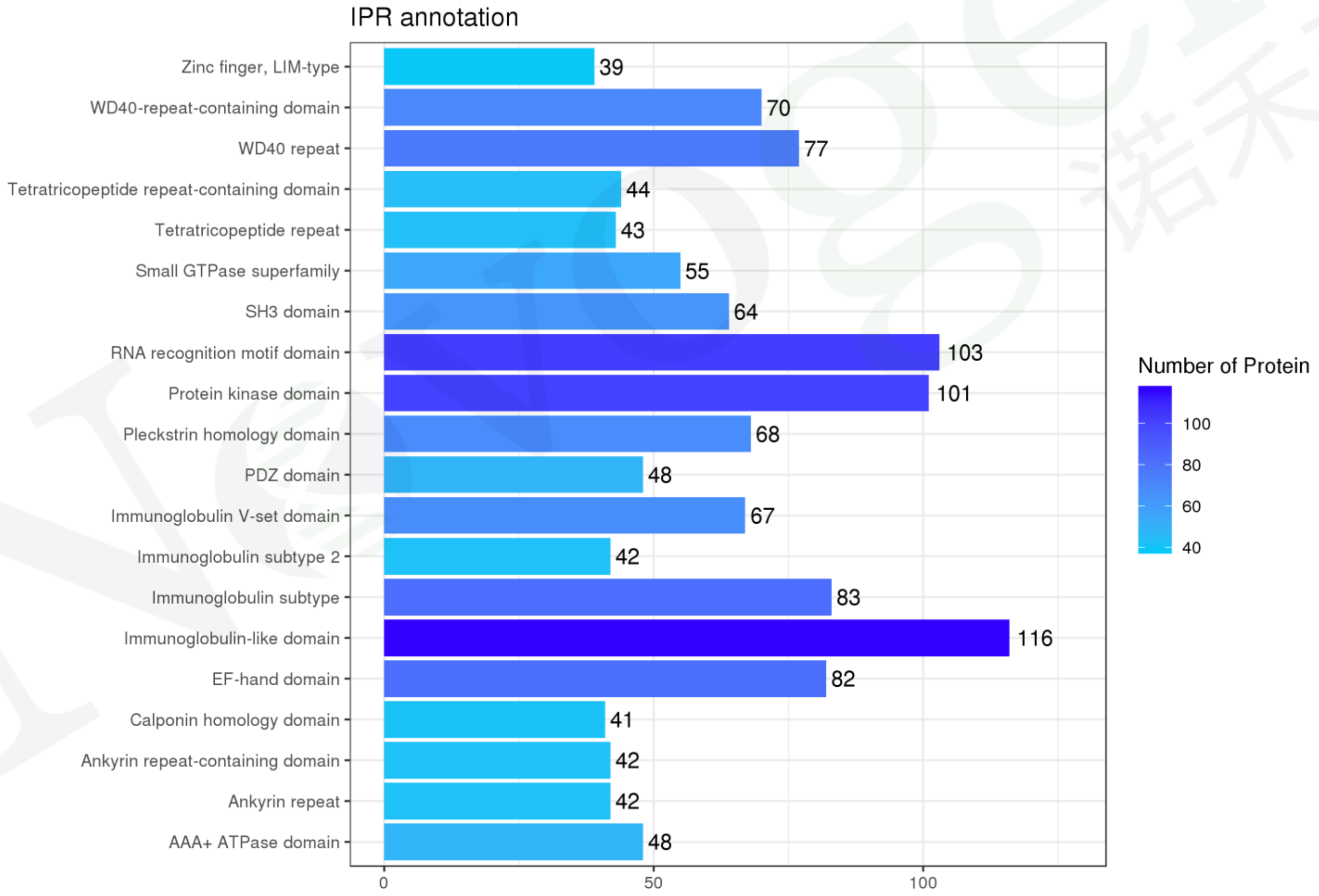

KEGG注释

KEGG是有关Pathway的主要公共数据库（http://www.genome.jp/kegg/），通过 Pathway 分析能确定蛋白质参与的最主要生化代谢途径和信号转导途径。基于 Pathway 的分析有助于更进一步了解其生物学功能。

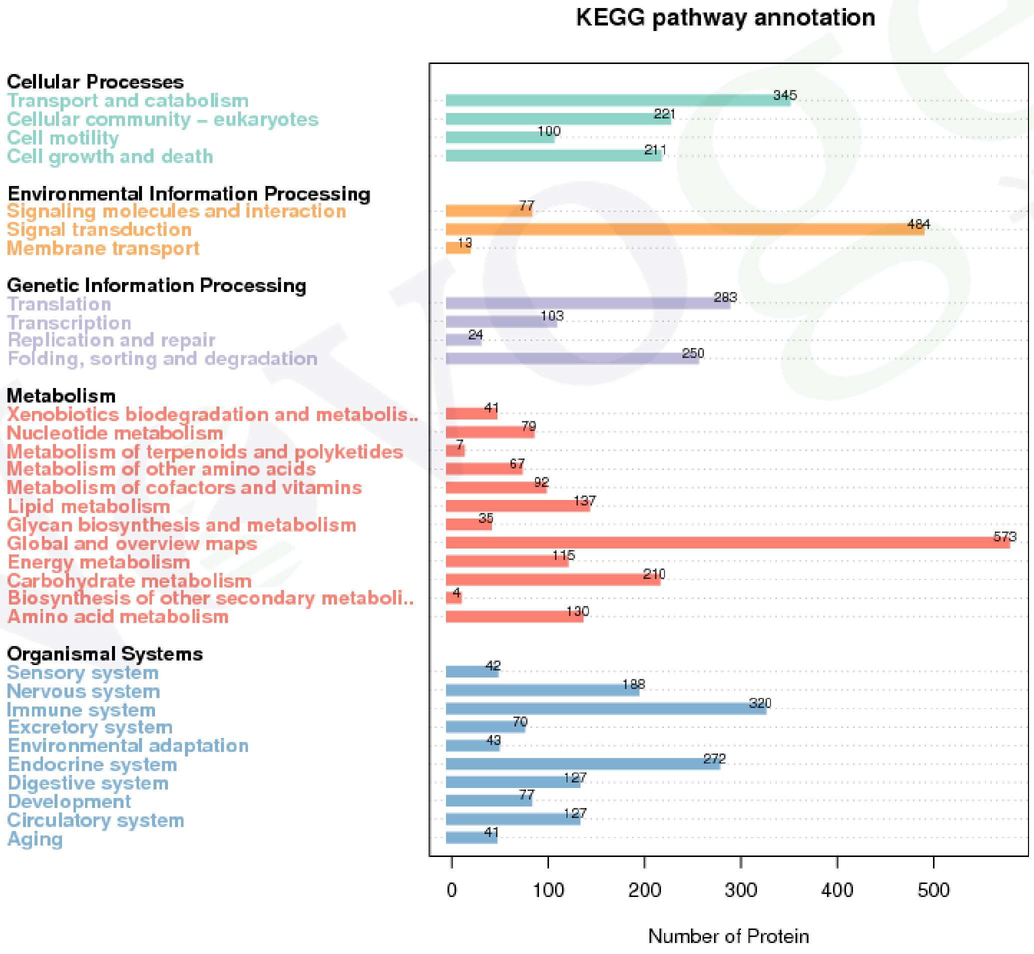

COG注释

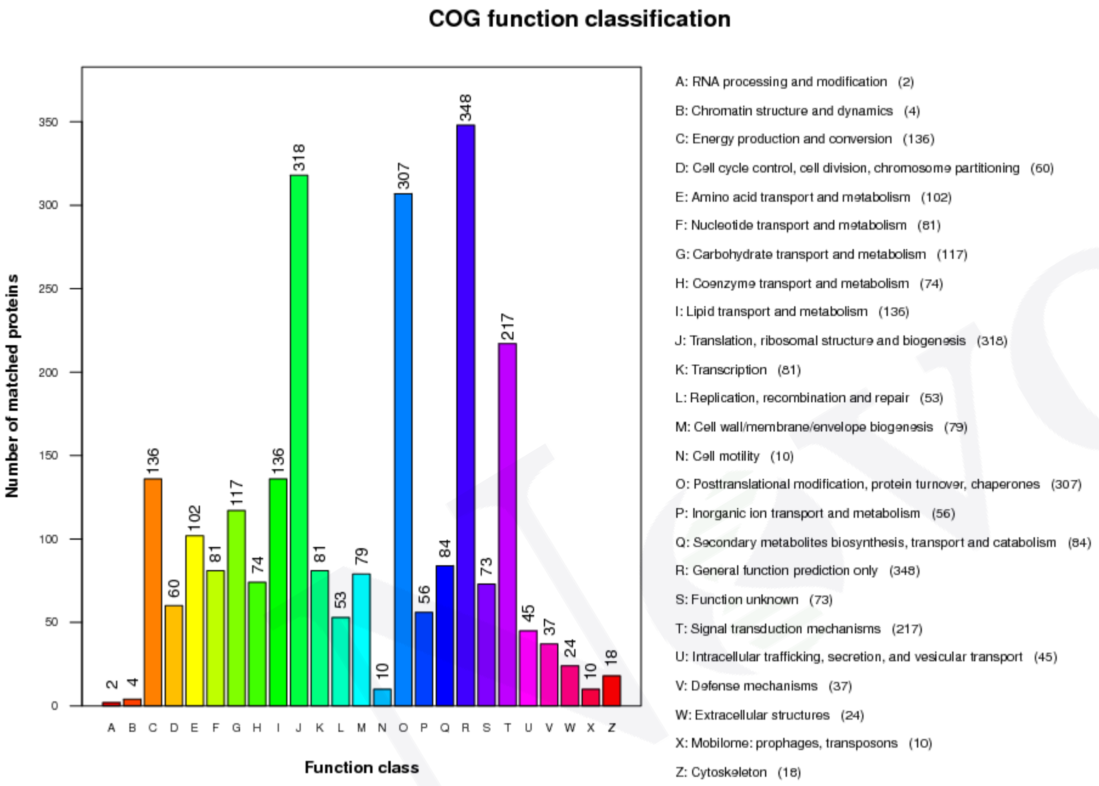

- COG（http://www.ncbi.nlm.nih.gov/COG/），全称是Cluster of Orthologous Groups of proteins（直系同源蛋白质簇）；
- COG数据库按照功能一共可以分为二十六类；
- 由NCBI创建并维护的蛋白数据库，根据细菌、藻类和真核生物完整基因组的编码蛋白系统进化关系分类构建而成。
- 通过比对可以将某个蛋白序列注释到某一个COG中，每一簇COG由直系同源序列构成，从而可以推测该序列的功能。

亚细胞定位

Novogene 诺禾致源

- 基于cell-mPLOC数据库；
- 将物种分为Animal、Euk、Plant、Gpos、Gneg、Viurs

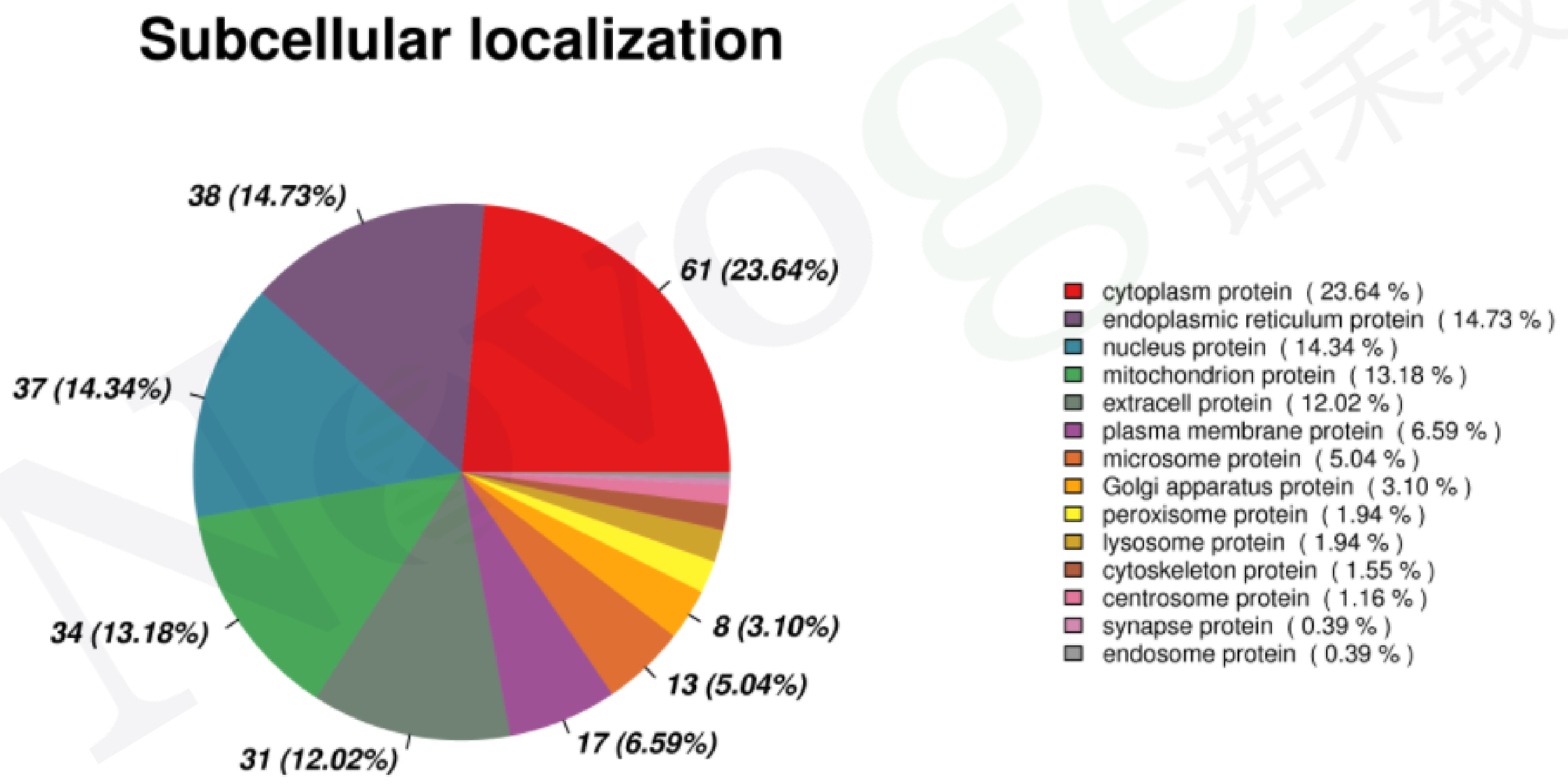

注释交付文件

- 0.raw.data
- 1.data_control
- 2.func.anno
- 3.ProtExprQuantification
- 4.DiffExprAnalysis
- 5.GOenrichment
- 6.KEGGenrichment
- 7.IPRenrichment
- 8.Subcellular_Diff
- 9.PPI
- results_dir
- results_readme

```
2.func.anno/
├── 2.1.GO
牋 ├── 2.1.GO_README.txt
牋 ├── GO.anno.go2protein.xls
牋 ├── GO.anno.pdf
牋 ├── GO.anno.png
牋 └── GO.anno.xls
├── 2.2.COG
牋 ├── 2.2.COG_README.txt
牋 ├── COG.anno.cog2protein.xls
牋 ├── COG.anno.pdf
牋 ├── COG.anno.png
牋 └── COG.anno.xls
├── 2.3.KEGG
牋 ├── 2.3.KEGG_README.txt
牋 ├── KEGG.anno.ko2protein.xls
牋 ├── KEGG.anno.pdf
牋 ├── KEGG.anno.png
牋 └── KEGG.anno.xls
├── 2.4.IPR
牋 ├── 2.4.IPR_README.txt
牋 ├── IPR.anno.ipr2protein.xls
牋 ├── IPR.anno.pdf
牋 ├── IPR.anno.png
牋 └── IPR.anno.xls
├── 2.5.Subcellar
牋 ├── 2.5.Subcellar_README.txt
牋 ├── Subcellular.localization_anno.xls
牋 ├── Subcellular.localization.pdf
牋 └── Subcellular.localization.png
└── 2.6.stat
    ├── 2.6.stat_README.txt
    ├── GO_KEGG_COG.anno.xls
    ├── stat.venn.png
    └── stat.venn.svg
```

- GO注释柱状图；
- GO注释表。
- COG注释柱状图；
- COG注释表。
- KEGG注释柱状图；
- KEGG注释表。
- IPR注释柱状图；
- IPR注释表。
- 亚细胞定位饼图；
- 亚细胞定位注释表。
- 功能注释韦恩图；
- 功能注释表。

Protein
Description
Gene
WJY1
WJY2
WJY3
CK1
CK2
CK3
GO_NUM
GO_Description
KEGG_Description
KEGG_EC
KEGG_ko
COG_Function_Description
COG_Function_Class
COG_Class_Description

2.3 蛋白定量结果

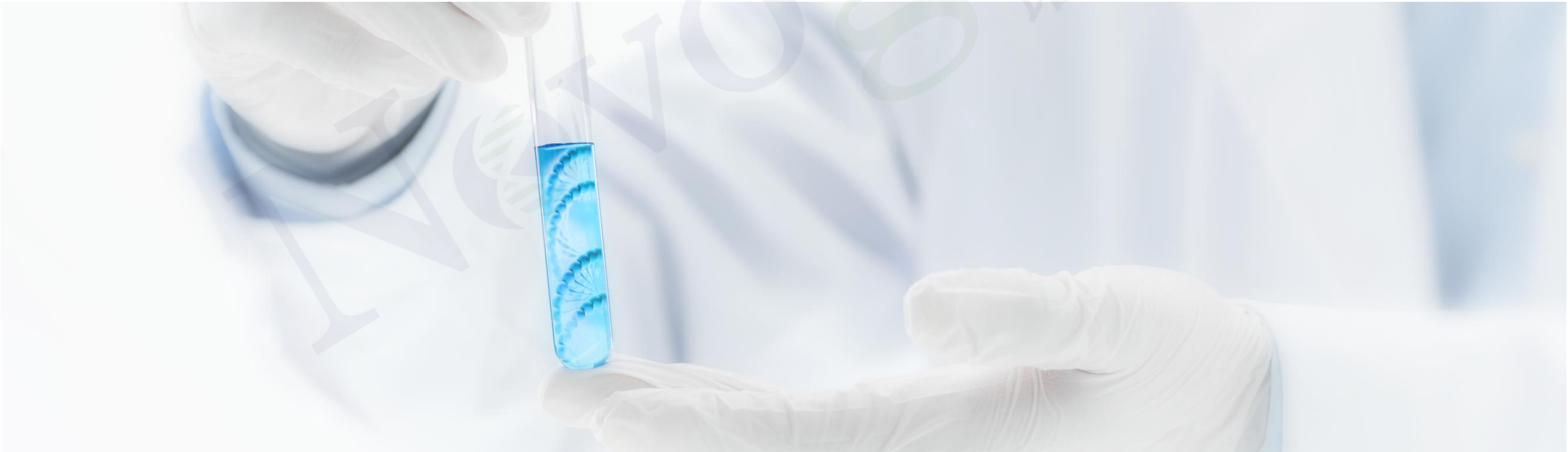

定量分析

蛋白定量结果

Protein	Description	Gene	M3_11B	M3_12B	M3_13B	M3_15B
ENSRNOP00000000025.7	heme binding protein 1	Hebp1	0.973831775700935	0.855807743658211	1.20240320427236	0.931108144192256
ENSRNOP00000000058.3	C-reactive protein	Crp	0.863336223571	1.08997532181685	1.09891282598546	1.00300140065364
ENSRNOP00000000064.1	cyclin G associated kinase	Gak	1.07159823549372	1.22192059721751	1.12385476756023	1.04207668815745

- Protein：蛋白在老师提供数据库中的编号；
- Description：蛋白在老师提供数据库中的描述信息（若数据库中无信息，则不展示）；
- Gene：蛋白在老师提供数据库中的对应基因名称（若数据库中无信息，则不展示）；
- 其他列为各个样品的相对定量信息

PCA分析

主成分分析（Principal component analysis, PCA）是将一组可能存在相关性的变量，通过正交变换转换为一组线性不相关变量的统计方法，转换后的这组变量即称为主成分。通过PCA分析从总体上反映各组样本之间的总体蛋白差异和组内样本之间的变异度大小。

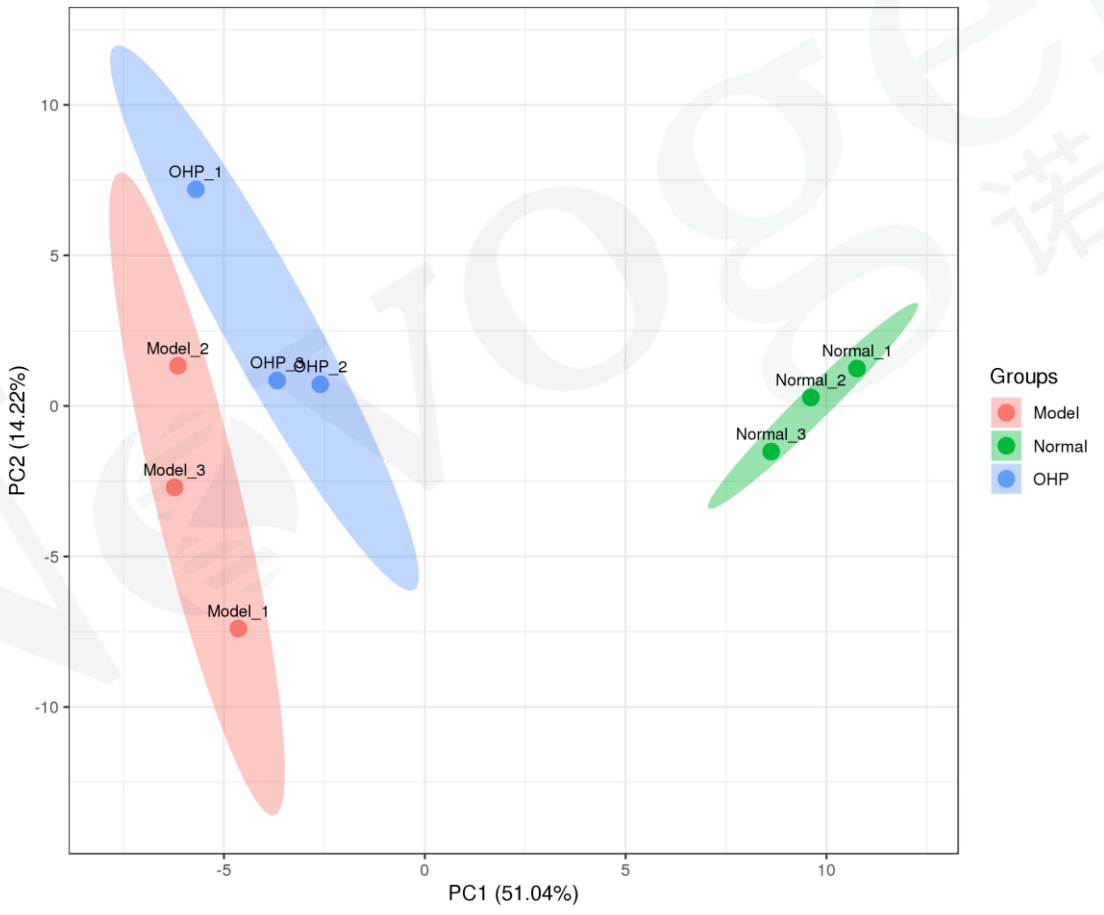

定量交付结果文件

- 0.raw.data
- 1.data_control
- 2.func.anno
- 3.ProtExprQuantification
- 4.DiffExprAnalysis
- 5.GOenrichment
- 6.KEGGenrichment
- 7.IPRenrichment
- 8.Subcellular_Diff
- 9.PPI
- results_dir
- results_readme

```
3.ProtExprQuantification/
├── 3.ProtExprQuantification_README.txt
├── all_sample.fa   鉴定到的蛋白序列
├── all_sample.xls  蛋白描述及定量值信息
├── PCA.pdf
├── PCA.png         PCA分析
└── Pep_pro.xlsx    蛋白、肽段的定量、修饰、可信度等信息
```

Protein	蛋白ID
Description	蛋白描述
Gene	gene id
A1	标准化的定量值
A2	
B1	
B2	

Header line description of worksheet "Protein"	
Protein FDR Confidence	蛋白可信度，High：FDR（strict）<0.01
Accession	蛋白ID
Exp. q-value	搜索时，其匹配度用 q 值来代表，值越小代表可信度越高
Sum PEP Score	所有 PSM 的 PEP 值负对数的加和
Coverage [%]	蛋白序列中氨基酸覆盖度
Peptides	鉴定到不同肽段的总数目
PSMs	肽段匹配到二级谱图的数目
Unique Peptides	特异性肽段数目
AAs	氨基酸数目
MW [kDa]	蛋白分子量
calc. pI	蛋白等电点
Score Sequest HT	Sequest HT 对蛋白匹配度的打分，分数越高可信度越高
Razor Peptides	与其他蛋白共享的多肽数目
Abundances (Normalized)	该蛋白的丰度标准化，校正实验偏差
Abundance	该蛋白的丰度值

2.4 差异蛋白分析

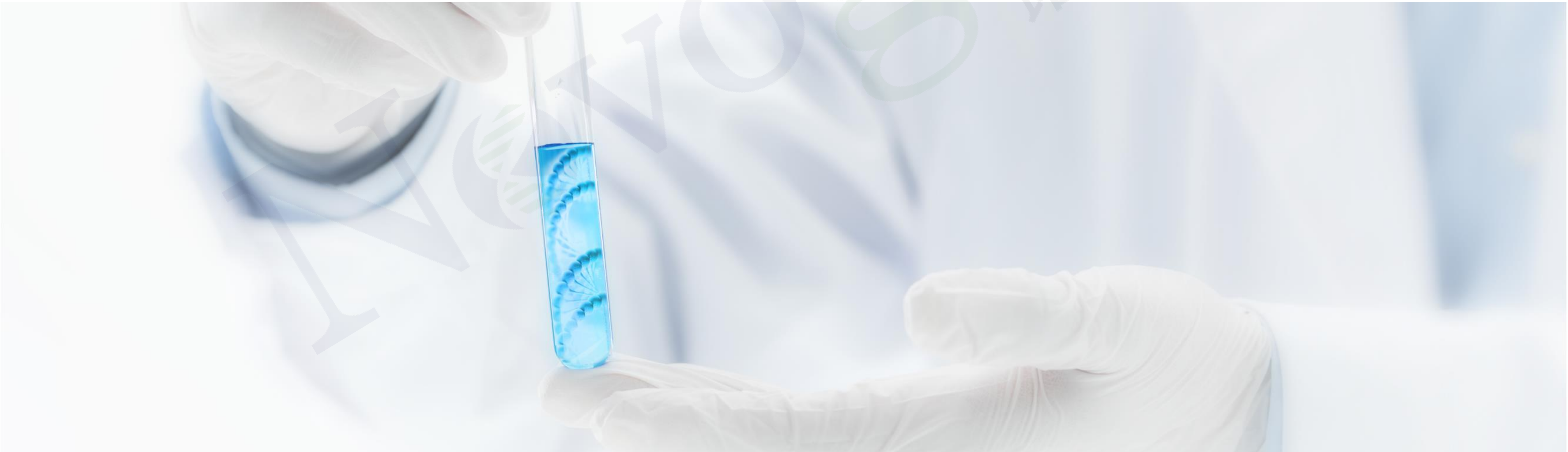

差异分析

- 蛋白差异分析首先挑出需要比较的样品对，将每个蛋白在比较样品对中的所有生物重复定量值的均值的比值作为差异倍数（Fold Change，FC）。
- 为了判断差异的显著性，将每个蛋白在两个比较对样品中的相对定量值进行了T-test检验，并计算相应的Pvalue（无重复时，用significant A算法计算P value）。以此作为显著性指标，默认Pvalue≤0.05

Compared Samples	Num. of Total Quant.	regulated Type	fold-change>1.2	fold-change>1.3	fold-change>1.5	fold-change>2.0
Obesity.vs.Control	4338	up-regulated	138	68	32	11
		down-regulated	64	17	4	1

如图：该项目选择当FC≥1.2，同时Pvalue≤0.05时，筛选上调表达蛋白，当FC≤0.83，同时Pvalue≤0.05时，筛选下调表达蛋白

差异火山图

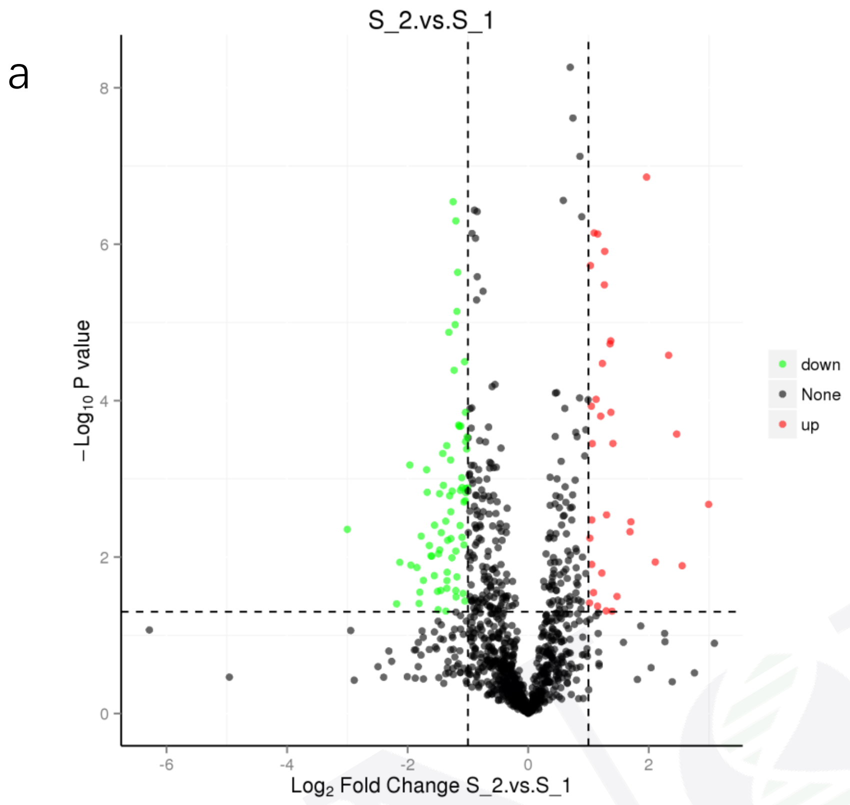

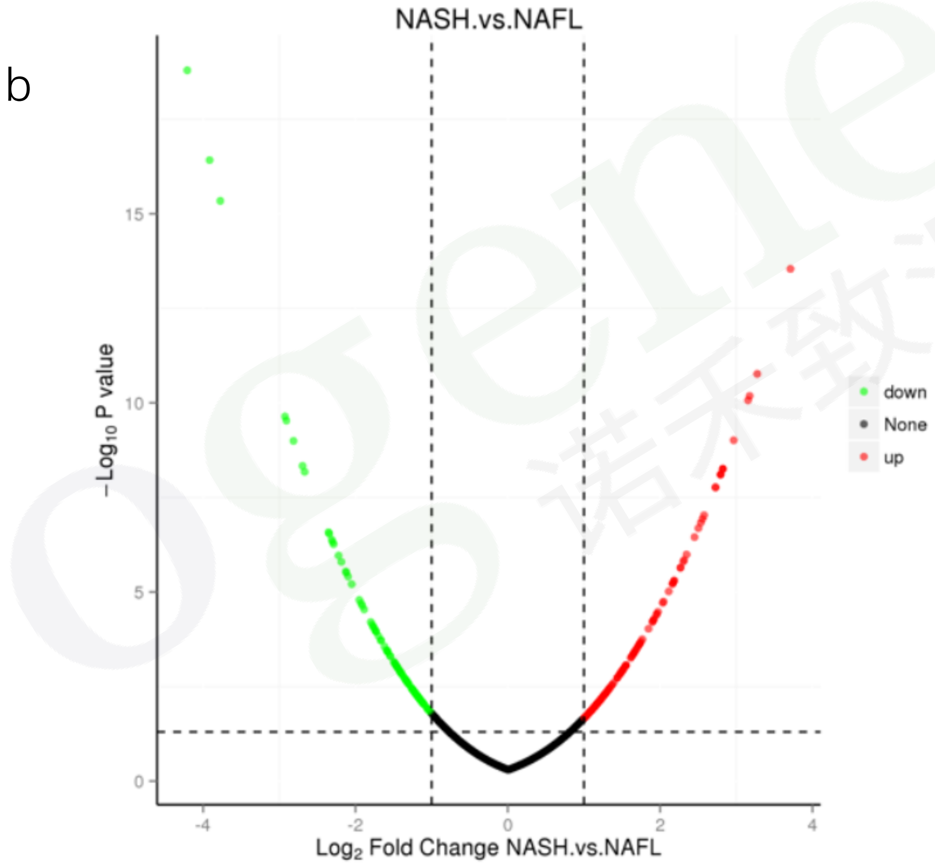

a.有生物学重复时，用T-test检验计算P value

b.无重复时，用significant A算法计算P value

差异蛋白质火山图：横坐标表示差异蛋白的差异倍数（log2值），纵轴表示Pvalue（-log10值），根据阈值（图中的虚线）筛选不同类型患者的差异蛋白质；黑色代表差异不显著的蛋白，红色代表显著上调蛋白，绿色代表显著下调蛋白。

差异聚类热图

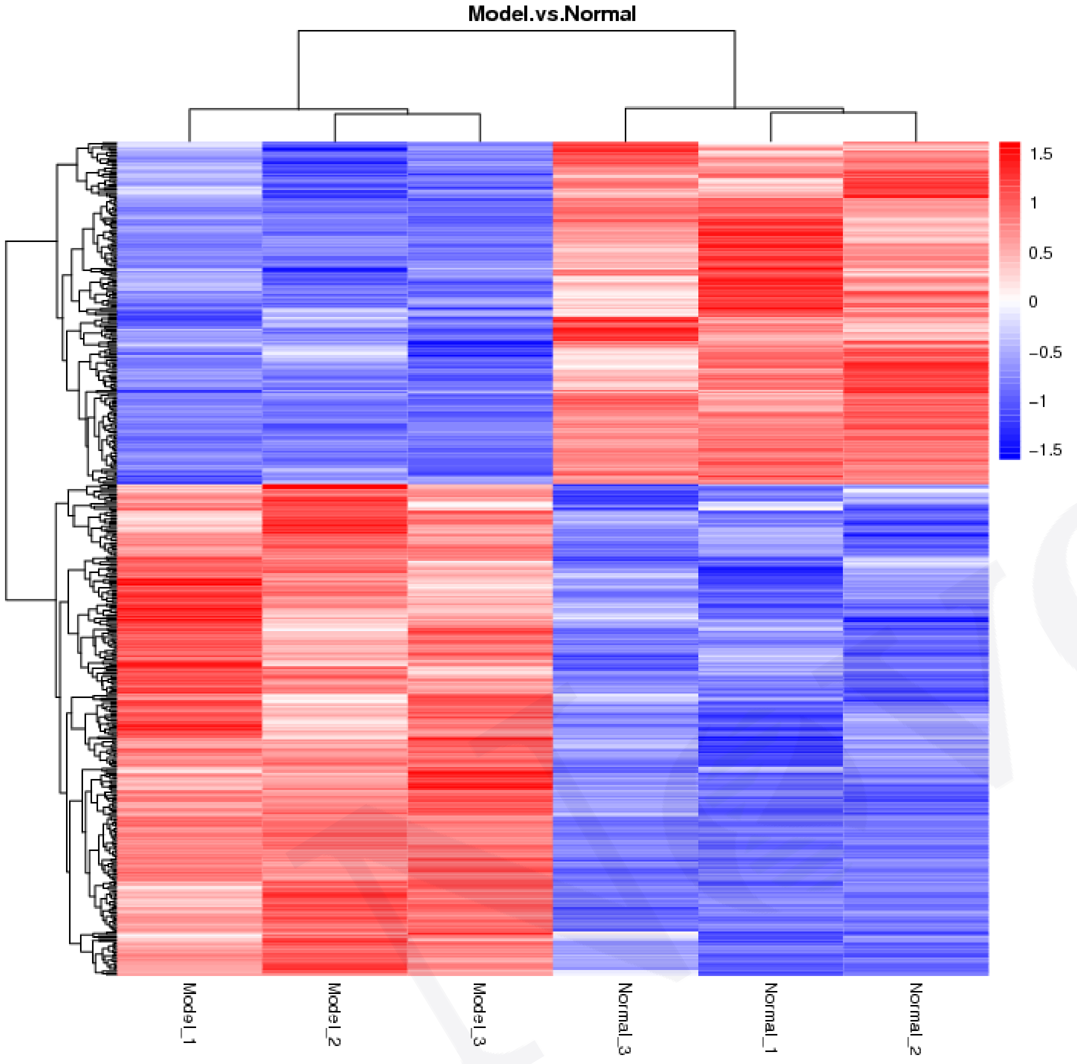

- 对每个样品中差异蛋白相对含量进行聚类分析；
- 利用聚类热图观察不同蛋白在不同样品间比较时的上调、下调情况；
- 每行进行了Z值校正，（观测值-行均值）/行标准差;
- 无重复时没有差异聚类热图。

纵向是样品的聚类，横向是蛋白的聚类，聚类枝越短代表相似性越高。横向的蛋白聚类可以得到表达模式一致的蛋白

表达水平聚类分析

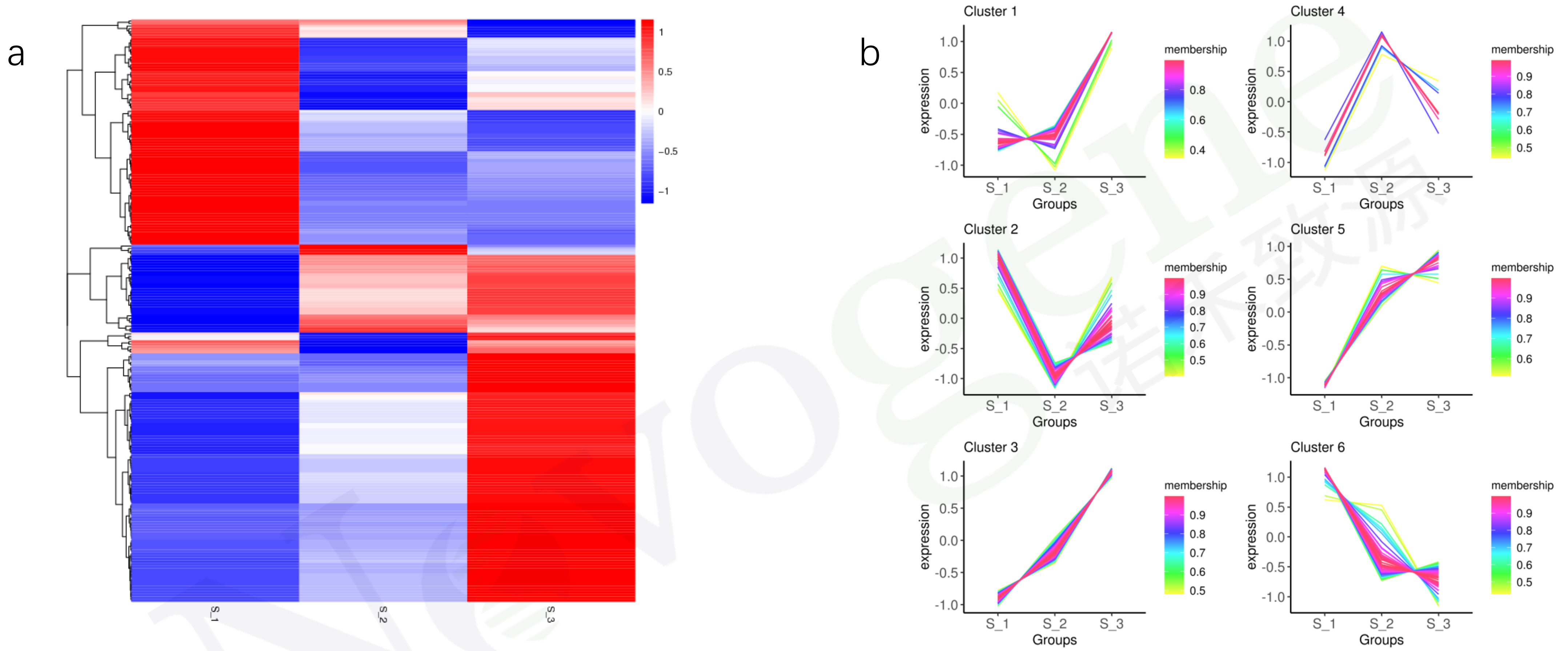

a. 差异蛋白表达水平聚类；纵向是组别的聚类，横向是蛋白的聚类，聚类枝越短代表相似性越高。从纵向聚类可以看出样品间蛋白含量的表达模式聚类。

b. 差异蛋白C-means聚类：根据蛋白的表达水平将蛋白分为若干类，横坐标为分组，纵坐标为表达量的Z值校正值，越大代表表达量越高，反之代表表达量越低。每条折线代表1个蛋白，颜色代表值的大小，值越大代表该蛋白在该分类中越接近平均水平。

差异分析交付文件

- 0.raw.data
- 1.data_control
- 2.func.anno
- 3.ProtExprQuantification
- 4.DiffExprAnalysis
- 5.GOenrichment
- 6.KEGGenrichment
- 7.IPRenrichment
- 8.Subcellular_Diff
- 9.PPI
- results_dir
- results_readme

```
4.DiffExprAnalysis/
├── 4.DiffExprAnalysis_README.txt
├── all_diff_prot_sig.xls
├── all_diff_prot.xls
├── cluster_heatmap.pdf
├── cluster_heatmap.png
├── cluster.png
├── cluster.svg
├── cluster.txt
├── Model.vs.Normal
牋 ├── Model.vs.Normal.diff_prot.xls
牋 ├── Model.vs.Normal.heatmap.detail1.pdf
牋 ├── Model.vs.Normal.heatmap.detail2.pdf
牋 ├── Model.vs.Normal.heatmap.detail.pdf
牋 ├── Model.vs.Normal.heatmap.pdf
牋 ├── Model.vs.Normal.heatmap.png
牋 ├── Model.vs.Normal.heatmap.xls
牋 ├── Model.vs.Normal.protein.xls
牋 ├── Model.vs.Normal.volcano.pdf
牋 └── Model.vs.Normal.volcano.png
```

all_diff_prot_sig.xls：差异蛋白的定量值、FC、P value信息

all_diff_prot.xls：所有蛋白的定量值、FC、P value信息

cluster.txt：每一个cluster中含有的蛋白ID

Model.vs.Normal.heatmap.detail1.pdf / detail2.pdf / detail.pdf：含有protein ID等的聚类热图

差异蛋白亚细胞定位分析

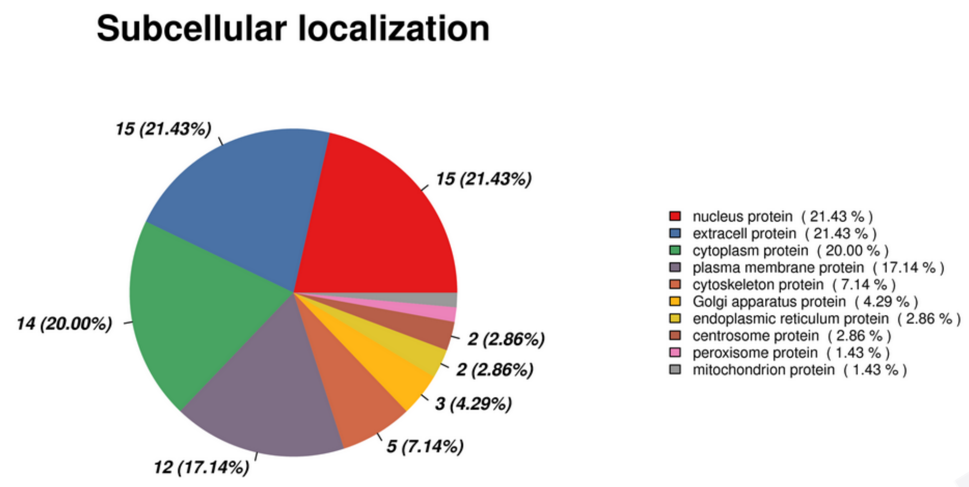

表8.差异蛋白亚细胞定位分析

对每个比较对的差异蛋白进行
亚细胞定位占比统计图分析

- 0.raw.data
- 1.data_control
- 2.func.anno
- 3.ProtExprQuantification
- 4.DiffExprAnalysis
- 5.GOenrichment
- 6.KEGGenrichment
- 7.IPRenrichment
- 8.Subcellular_Diff
- 9.PPI
- results_dir.html
- results_readme.pdf

- MHC_MI.vs.KO_MI.Subcellular_diff.pdf
- MHC_MI.vs.KO_MI.Subcellular_diff.png
- MHC_MI.vs.KO_MI.Subcellular_diff.xls

➢ 差异蛋白亚细胞定位饼图；
➢ 差异蛋白亚细胞定位注释表

差异蛋白互作网络图

方法：利用StringDB蛋白质互作数据库(http://string-db.org/)进行鉴定蛋白的互作分析，差异蛋白的序列与提取出的序列进行blast比对，得出相应的互作信息，构建网络图。结果文件中提供了蛋白互作数据的文件，可以直接导入 Cytoscape 软件进行可视化编辑。

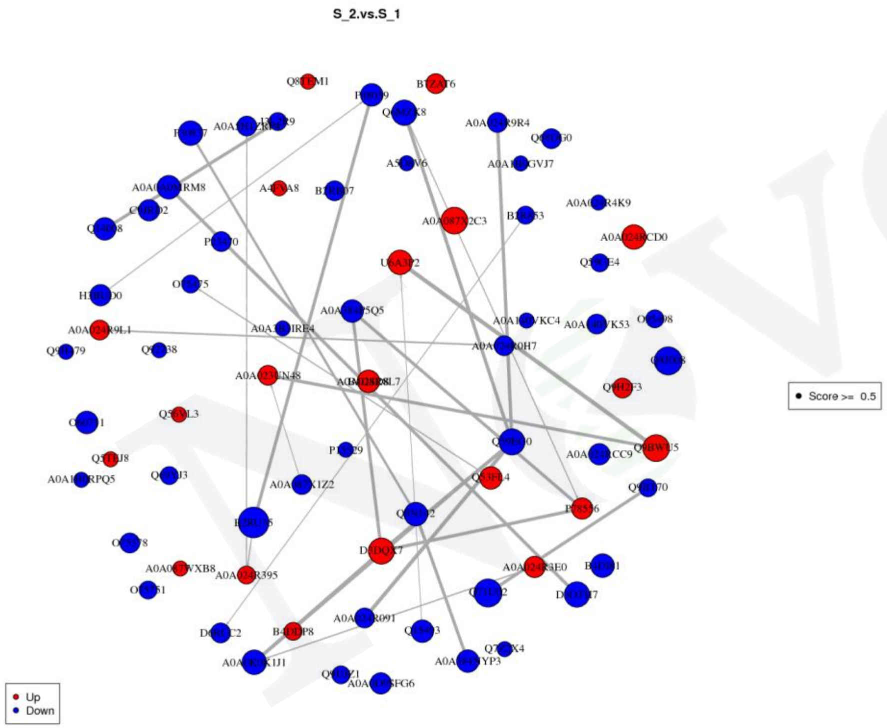

蛋白互作网络图:网络中每个节点表示一个蛋白，节点大小表示与之互作蛋白的个数，节点越大说明与之互作的蛋白越多；节点的颜色表示此蛋白在比较对的表达水平，红颜色代表此蛋白显著高表达，绿颜色代表此蛋白显著低表达

差异蛋白PPI结果文件

- 0.raw.data
- 1.data_control
- 2.func.anno
- 3.ProtExprQuantification
- 4.DiffExprAnalysis
- 5.GOenrichment
- 6.KEGGenrichment
- 7.IPRenrichment
- 8.Subcellular_Diff
- 9.PPI
- results_dir
- results_readme

- network_src
- A_INS.vs.B_SEN.Net_down_PPI
- A_INS.vs.B_SEN.Net_PPI
- A_INS.vs.B_SEN.Net_up_PPI
- A_INS.vs.B_SEN.StringDB_cut500
- A_INS.vs.B_SEN.StringDB_cut500

两两互作蛋白ID及其得分值

prot1	prot2	score
B2R9U2	B2R6R6	224
B2R9U2	E5KT65	179
B2R9U2	Q00688	310
B2R9U2	O95433	582
B2R9U2	Q05DF2	173
B2R9U2	P22087	297

2.5 功能富集分析

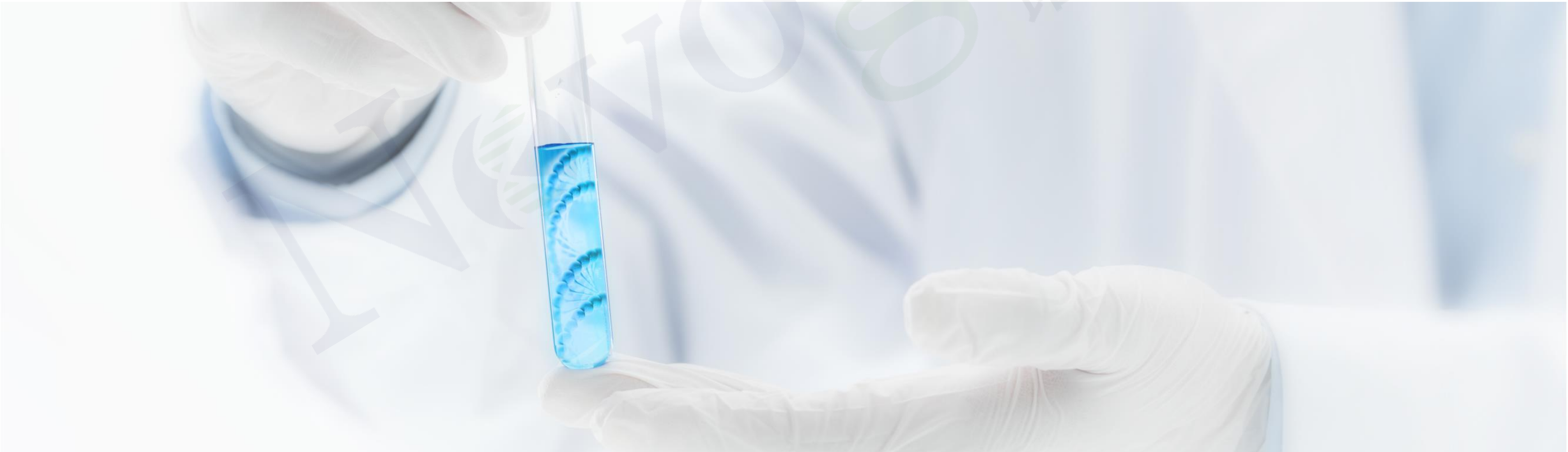

差异富集分析

- 功能富集
  - GO富集
    - 富集柱状图
  - KEGG富集
    - 富集气泡图
    - 富集通路图
  - IPR富集
    - 富集气泡图

GO富集分析

Novogene 诺禾致源

表5.1 GO富集结果

a

GO_ID	GO_Term	GO_Class	Pvalue	AdjustedPv	x	y	n	N	GOlevl	
GO:0005515	protein binding	MF	0.000332363805001334	0.10220187003791	104	2148	158	4170	3	A0A024R091 A0A024R0H7 A0A024R3
GO:0005739	mitochondrion	CC	0.0133823197069831	0.219887126383981	5	38	158	4170	5	
GO:0030145	manganese ion binding	MF	0.0193546921565625	0.219887126383981	2	6	158	4170	7	
GO:0098800	inner mitochondrial membrane protein complex	CC	0.0193546921565625	0.219887126383981	2	6	158	4170	4	
GO:0008519	ammonium transmembrane transporter activity	MF	0.0378896882494005	0.219887126383981	1	1	158	4170	7	

表格的详细内容如下： (1) GO_ID：GO标识号； (2) GO_Term：GO标识号功能的描述； (3) GO_Class：GO类别（MF,CC,BP）； (4) Pvalue：富集分析的Pvalue ； (5) AdjustedPv：校正后的Pvalue ； (6) x：与该GO相关的差异蛋白的数目； (7) y：与该GO相关的背景（所有）蛋白的数目； (8) n：GO注释的差异蛋白数目； (9) N：GO注释的背景（所有）蛋白的数目； (10) GOlevl：该term在GO数据库的层级数； (11) ProtID：蛋白ID ；

b

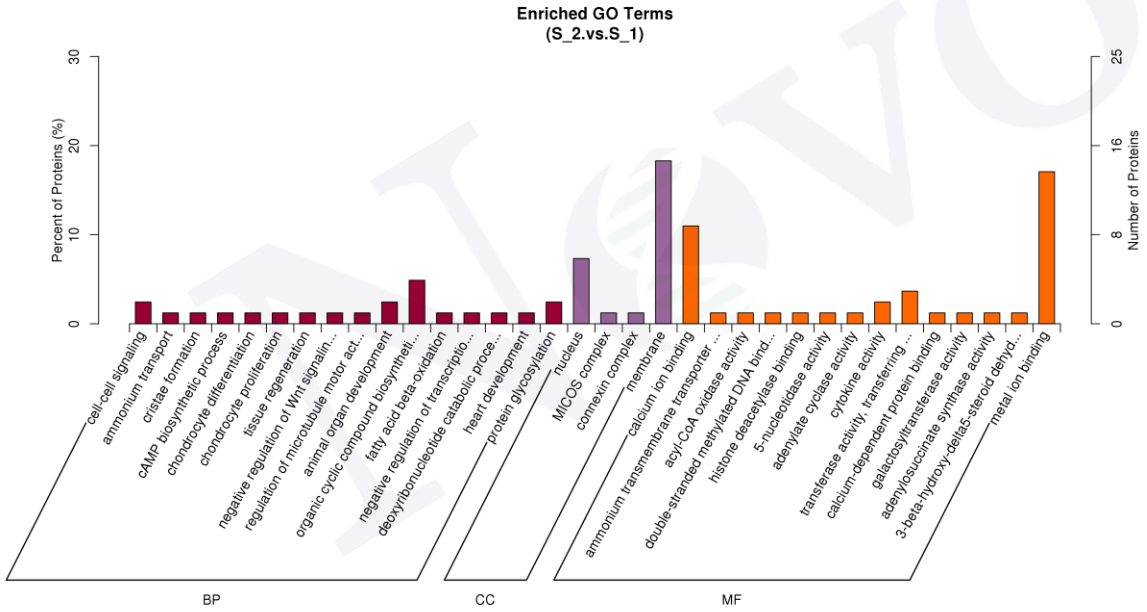

a. GO富集结果（x/y＞n/N即认为被富集到该通路）
b.图中展示了GO三个类别中的富集结果，纵坐标的百分比代表表表中的x/n,另一个纵坐标表示对应通路富集到的蛋白数。

KEGG富集分析

表6.1 KEGG富集结果

a

MapID	MapTitle	Pvalue	AdjustedPv	x	y	n	N	
map05143	African trypanosomiasis	0.000225001269839023	0.0261001473013267	16	280	56	2352	A0A024R3E0 A0A5C2G5Z3 A0A5C2G667 A0A5C2G6B6 A0A5(
map05414	Dilated cardiomyopathy (DCM)	0.000666935374265393	0.0334786694284923	16	299	56	2352	A0A5C2G5Z3 A0A5C2G667 A0A5C2G6B6 A0A5C2G883 A0.
map05323	Rheumatoid arthritis	0.000865827657633421	0.0334786694284923	16	304	56	2352	A0A024R3E0 A0A5C2G5Z3 A0A5C2G667 A0A5C2G6B6 A0A5
map05340	Primary immunodeficiency	0.00123117130301796	0.0357039677875207	14	255	56	2352	A0A5C2G5Z3 A0A5C2G667 A0A5C2G6B6 A0A5C2(
map04072	Phospholipase D signaling pathway	0.00178135594879095	0.035785759741347	15	290	56	2352	A0A0D9SFG6 A0A5C2G5Z3 A0A5C2G667 A0A5C2G6B6 A

b

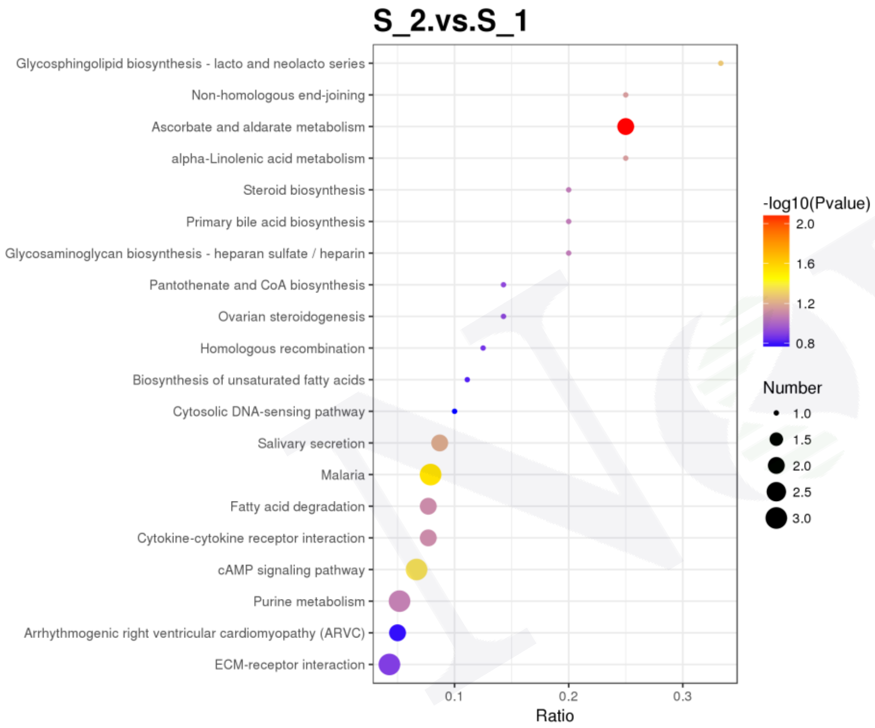

c

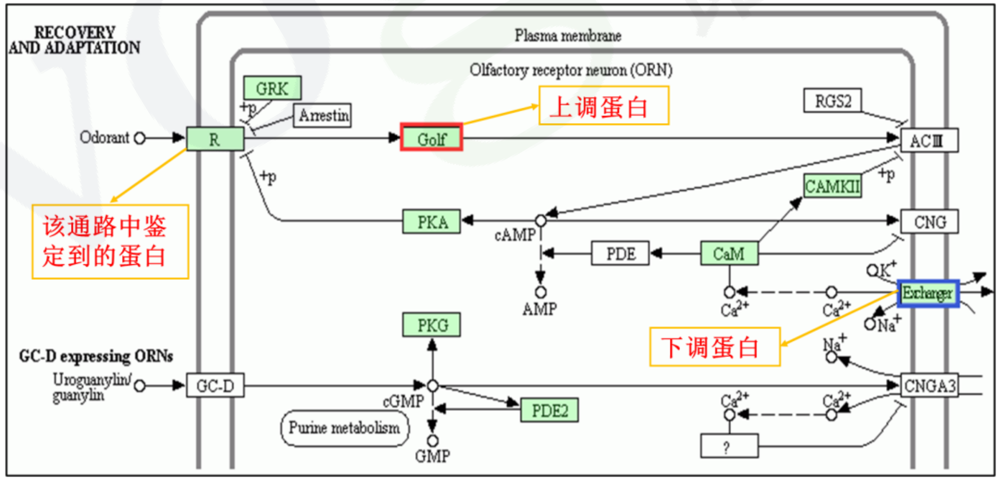

a. KEGG富集结果
b. KEGG富集气泡图：横坐标值越大，说明在该通路中差异蛋白富集程度越高。点的颜色代表超几何检验的Pvalue值，点的大小表示在该通路中差异蛋白数目的多少。
c. KEGG通路图：绿色底色框内为鉴定出的总蛋白，蓝色框标记的是下调差异蛋白，红色框标记的是上调差异蛋白，黄色框标记的是通路中该功能对应的多个蛋白中既有上调差异蛋白、也有下调差异蛋白。

IPR富集分析

横坐标：相应结构域中差异蛋白的数目与鉴定出的总蛋白数目的比值，值越大，说明在该结构域中差异蛋白富集程度越高。

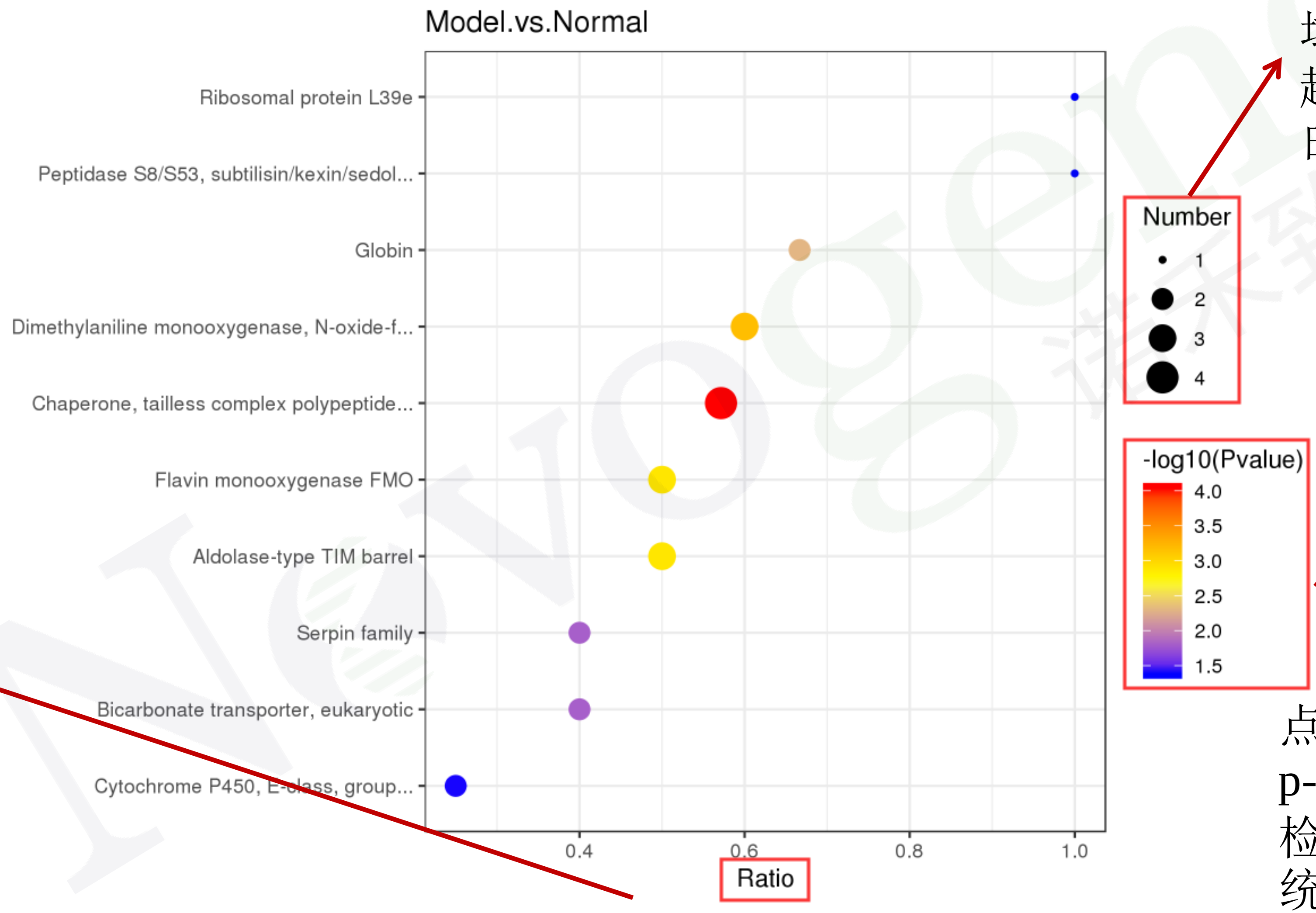

点的大小代表相应结构域中差异蛋白的数目，越大，该通路内差异蛋白就越多。

点的颜色：超几何检验的p-value值，值越小，说明检验的可靠性越大、越具统计学意义。

富集结果文件

Novogene 诺禾致源

- 0.raw.data
- 1.data_control
- 2.func.anno
- 3.ProtExprQuantification
- 4.DiffExprAnalysis
- 5.GOenrichment
- 6.KEGGenrichment
- 7.IPRenrichment
- 8.Subcellular_Diff
- 9.PPI
- results_dir
- results_readme

- UP
- DOWN
- ALL

- pathway
- A.vs.B.KEGG_Enrich.scatterplot
- A.vs.B.KEGG_Enrich.scatterplot

富集气泡图

- A.vs.B.KEGG_Enrich

富集注释表

MapID	MapTitle	Pvalue	AdjustedPv	x	y	n	N	EnrichDirect	ProtID
map04974	Protein digestion and absorption	0.00291412057389857	0.22451973508465	9	45	184	2670	Over	A0A024R5L0 A0A384MDP3 B7ZLK3 B7ZW00 D9ZGF2 P12109 P25940 Q05707 Q9BUM6
map04978	Mineral absorption	0.00460465555716721	0.22451973508465	5	17	184	2670	Over	A0A140VJP8 A0A384MDR3 E5RIM7 Q6NS36 Q53TN4
map00010	Glycolysis / Gluconeogenesis	0.00460676109221726	0.22451973508465	9	48	184	2670	Over	A0A2U3TZU2 P00338 P06733 P07195 P60174 Q53G35 V9HWF4 V9HWN7 B2R7D2

原始raw文件

- 0.raw.data
- 1.data_control
- 2.func.anno
- 3.ProtExprQuantification
- 4.DiffExprAnalysis
- 5.GOenrichment
- 6.KEGGenrichment
- 7.IPRenrichment
- 8.Subcellular_Diff
- 9.PPI
- results_dir
- results_readme

质谱下机文件

```
$ tree 0.raw.data/
0.raw.data/
├── 0.1.raw_file
牋 └── 0.1.raw_file_README.txt
└── 0.2.match_result
    ├── 0.2.match_result_README.txt
    ├── HFX1_FFRA19H0021_PeptideGroups.txt
    └── HFX1_FFRA19H0021_Proteins.txt
```

搜库得到的肽段信息

搜库得到的蛋白信息

3.蛋白数据上传

iProx 和 ProteomeXchange 是两个常用的上传蛋白质组学质谱数据的存储平台

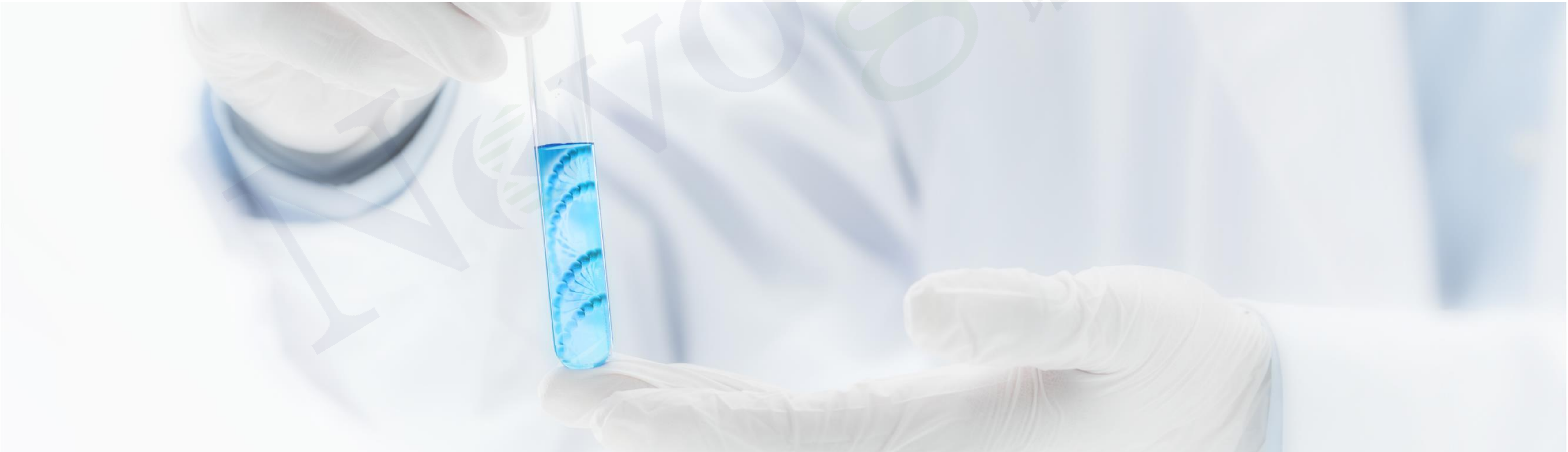

Step1: 注册账号

1、进入iProx主页：

https://www.iprox.org/

2、注册账号填写信息：

➢ 选择主页右上角的“Register”

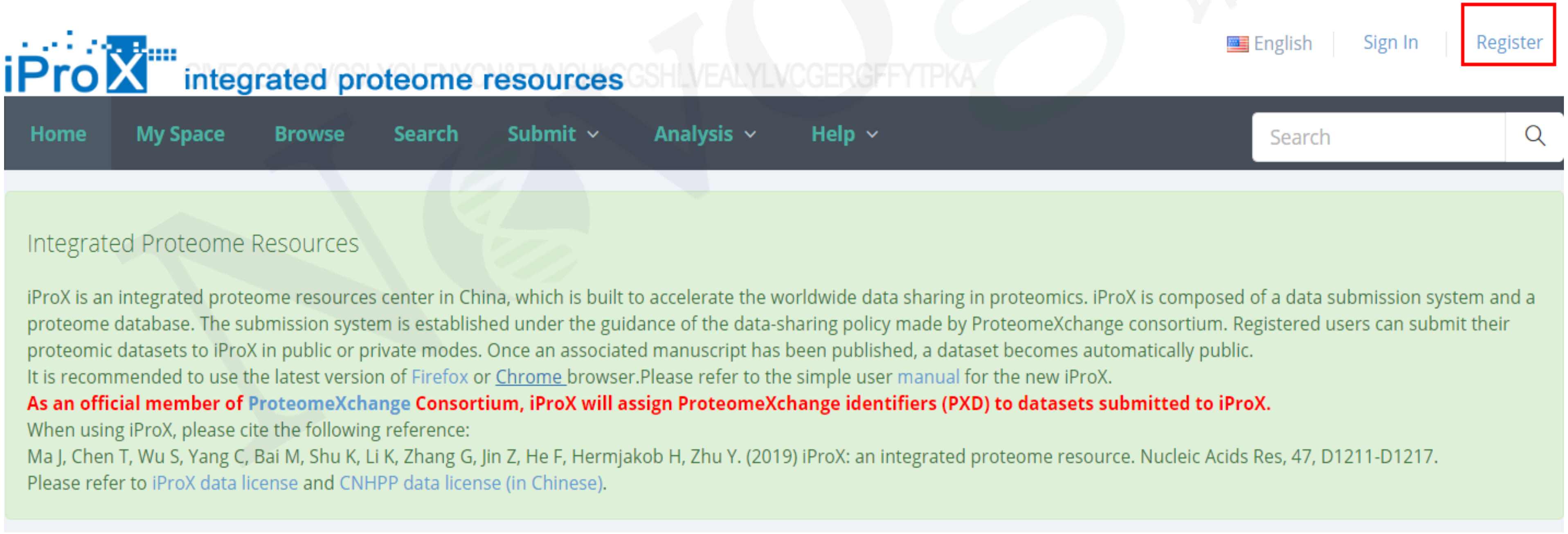

➢ 新出现的页面中填写个人信息，依次填完2、3步；

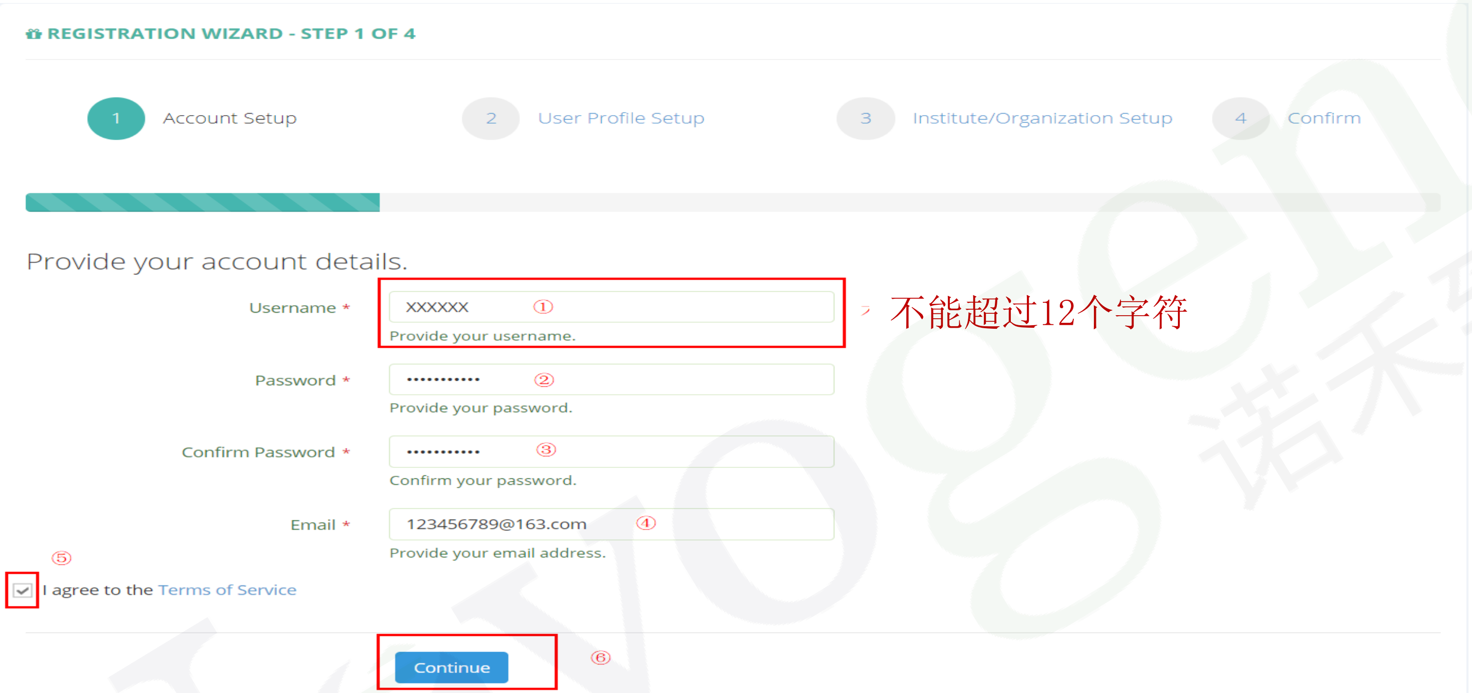

➢ 第4步确定填写的信息，核实填写内容正确时，点击“Submit”

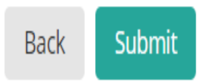

Step2: 激活账号

- 去填写的邮箱，激活账号；
- 点击邮件正文的的链接，完成邮件激活；

Thanks for registering iProX!

Please click the following link to activate your account in 48 hours.

https://www.iprox.org/page/URV001.html?userName=december&password=dai88336539

Following is the same content in Chinese.

以下是与上面英文信息相同的中文信息。

感谢您注册iProX!

请点击下面链接激活账号，链接有效期为48小时，超过期限请重新注册。

https://www.iprox.org/page/URV001.html?userName=december&password=dai88336539

- 出现这个提示，注册就完成了，就可以在网站进行登陆。

Email has been activated, please log in

Step3: 登录账号

1、点击右上角“Sign In”；

2、填入用户名、密码、验证码，登陆个人账号

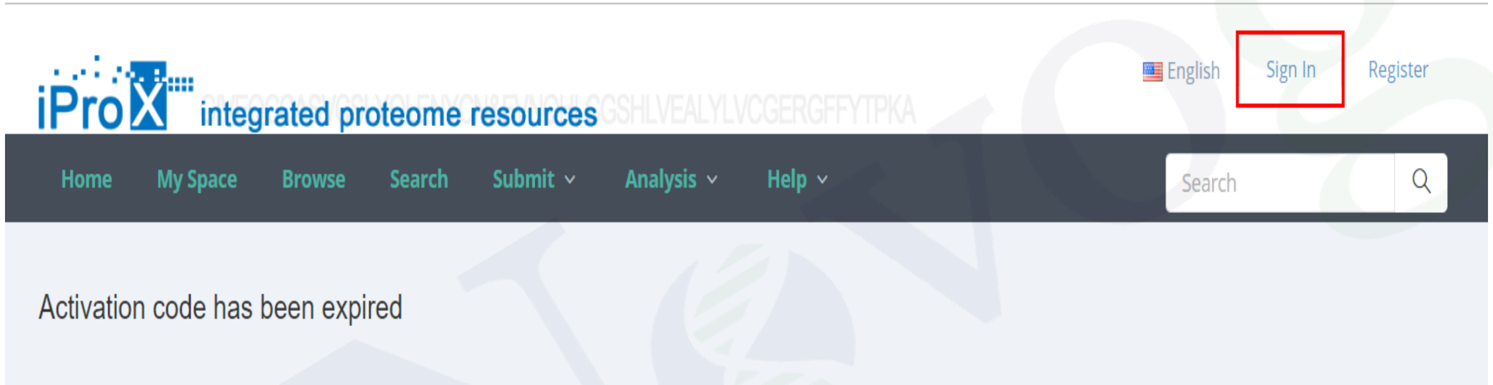

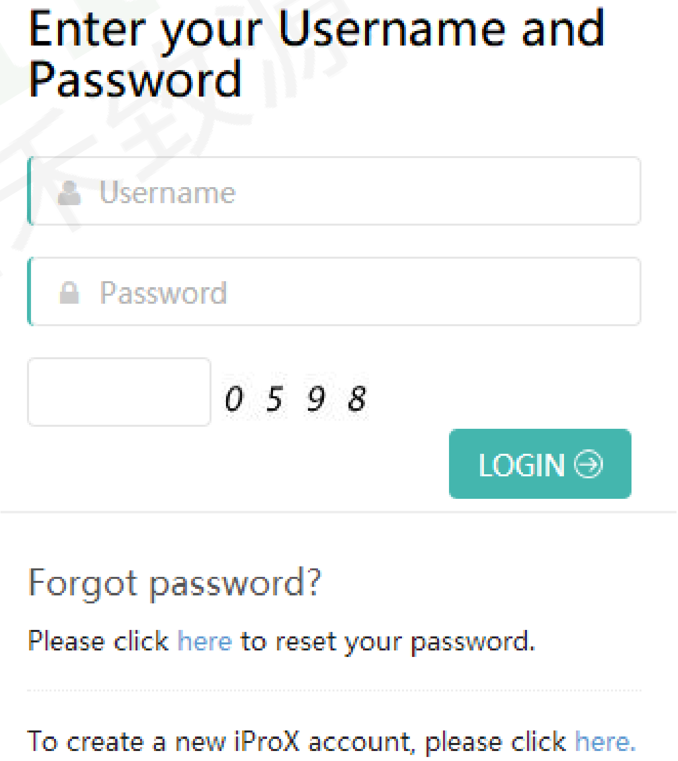

Step4: 提交数据

- 点击导航栏——My Space；
- 左部Project Tree里。点击“**Project**”；
- 点击右侧“**New SubProject**”，填写项目信息。

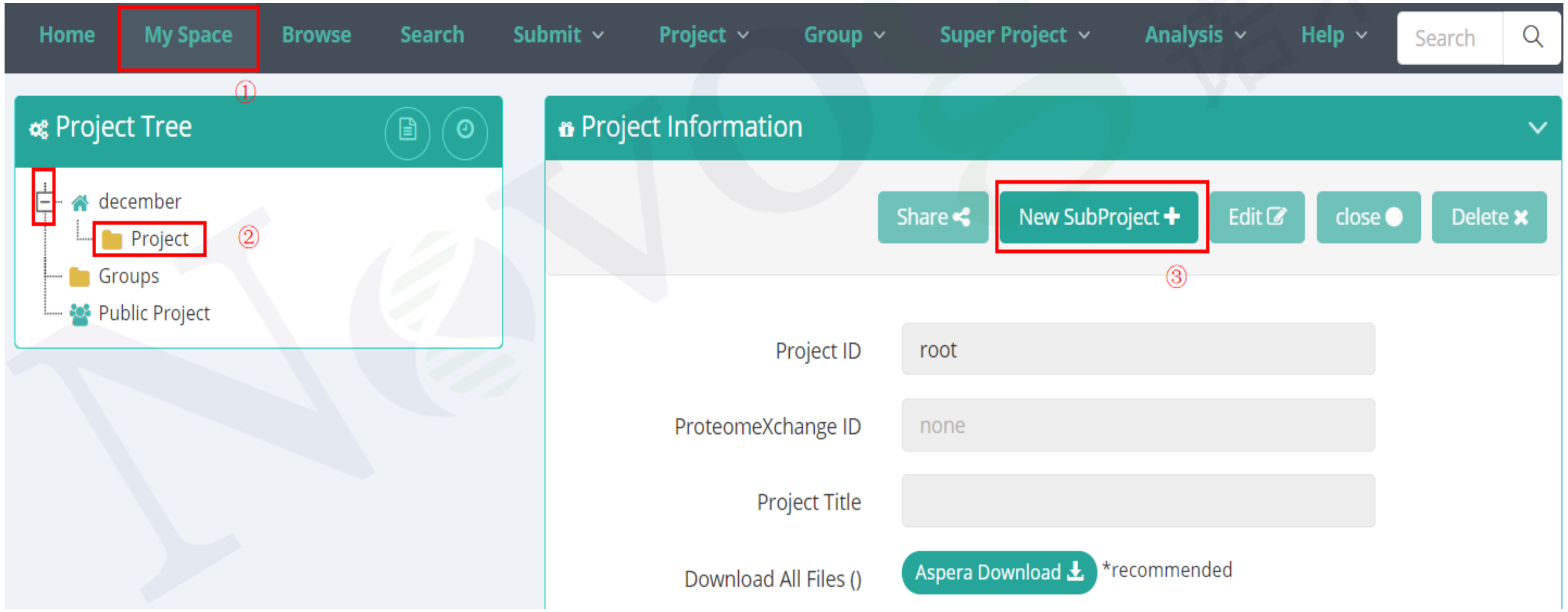

1 、Add New Project 添加新项目

Novogene 诺禾致源

Add New Project

Project ID: IPX0001717000 — Add link — 不
Project ID will be generated automatically.
Provide external links f

ProteomeXchange ID

Project Title * — 项目名称
Provide title of the project (required).

Project Tag (key words) * — 项目标签
Provide key words of the project (recommended).

Description * — 项目描述（不少于50个
Provide description of the project (recommended).

Institution * — 机构名
Provide your institution (required).

Submitter * — Add New + — 一般自动生成，如没有，可点击"Add New+"添加，填入后"save"

Name	Email	Institution	Edit	Delete
december			Edit	Delete

Lab Head * — Add New + — 点击"Add New+"添加，填入后"save"

Name	Email	Institution	Edit	Delete
			Edit	Delete

Publication — Add New +

PubMed ID	References	Edit	Delete
No data available in table			

Access Level * — Public — 三个选项，"Public"公开、"Plan to public"计划公开（在后面提示的日期栏选择将要公开的日期）、"Private"保密。
Data release note: the data will be released to public when the paper associated with this iProX ID is published, no matter 'Plan to public' or 'Private' is chosen here.

2、 Add New Subproject 添加新子项目

Add New SubProject

Subproject ID
Subproject ID will be generated automatically.

ProteomeXchange ID

Subproject Title * — 项目名称
Provide title of the subproject (required).

Subproject Tag
Provide key words of the subproject (recommended).

Species * — Homo sapiens — Choose — + New — 通过"choose"选择物种
Provide species information for your dataset (required).

MS instrument * — Q Exactive HF — ≡more — Add New MS instrument + — 质谱仪型号
The instrument name is required, and your can click the 'More' option to provide further instrument information.

没有标注选择为可选内容，根据具体情况选择填与不填

3、 Upload Data File：上传文件

Novogene 诺禾致源

目前大多采用**Partial submission**的形式来上传数据。

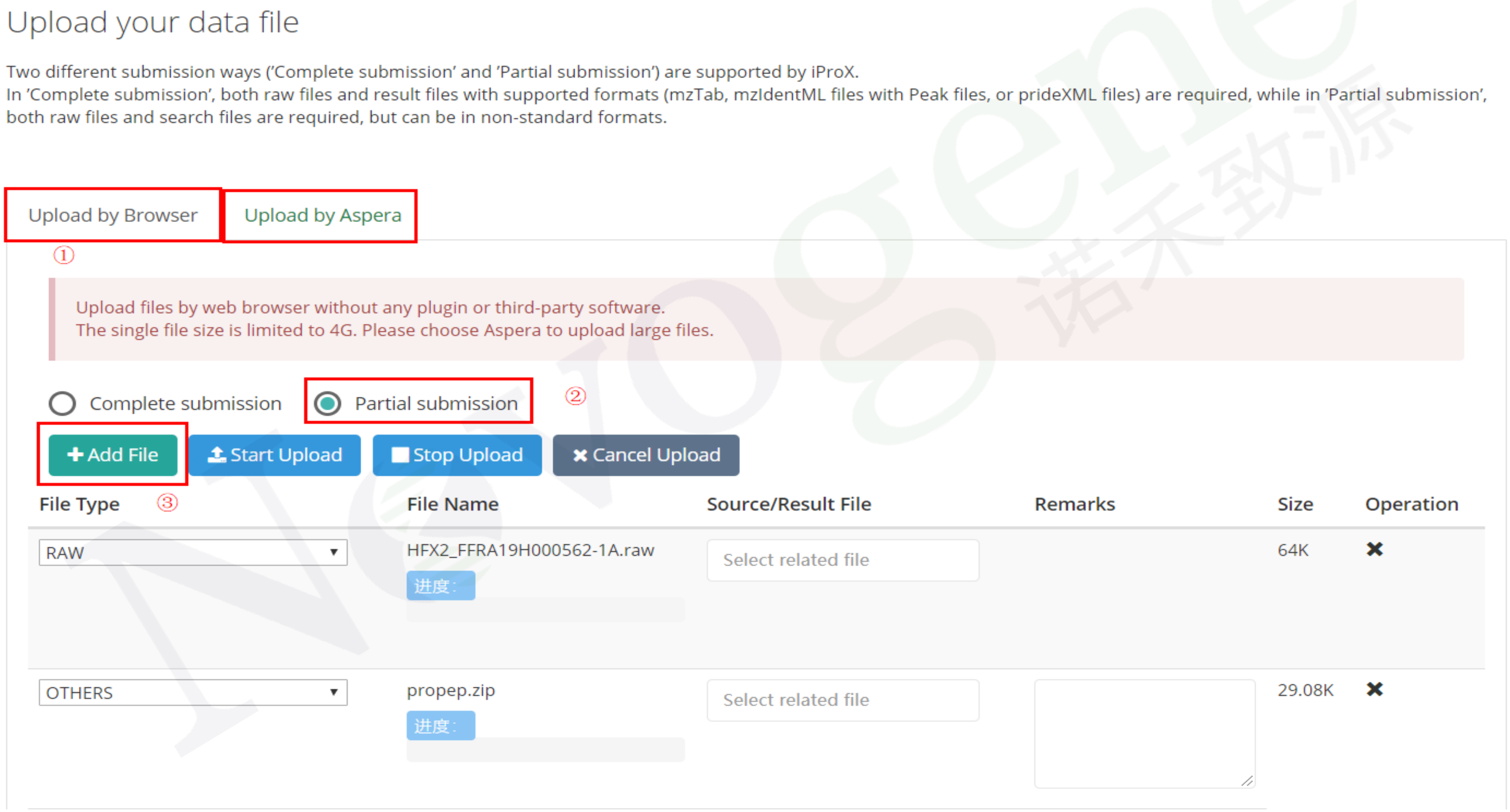

- Raw文件来自释放文件夹Raw_file中*.raw文件；
- Search file 文件来自Match_result，将*PeptideGroups.txt/*Proteins.txt文件准备好后，
- 点击“Add File”按钮，选中要上传的文件；
- 如果有必要需要在file type一栏的下拉菜单中修改一下“file type”。

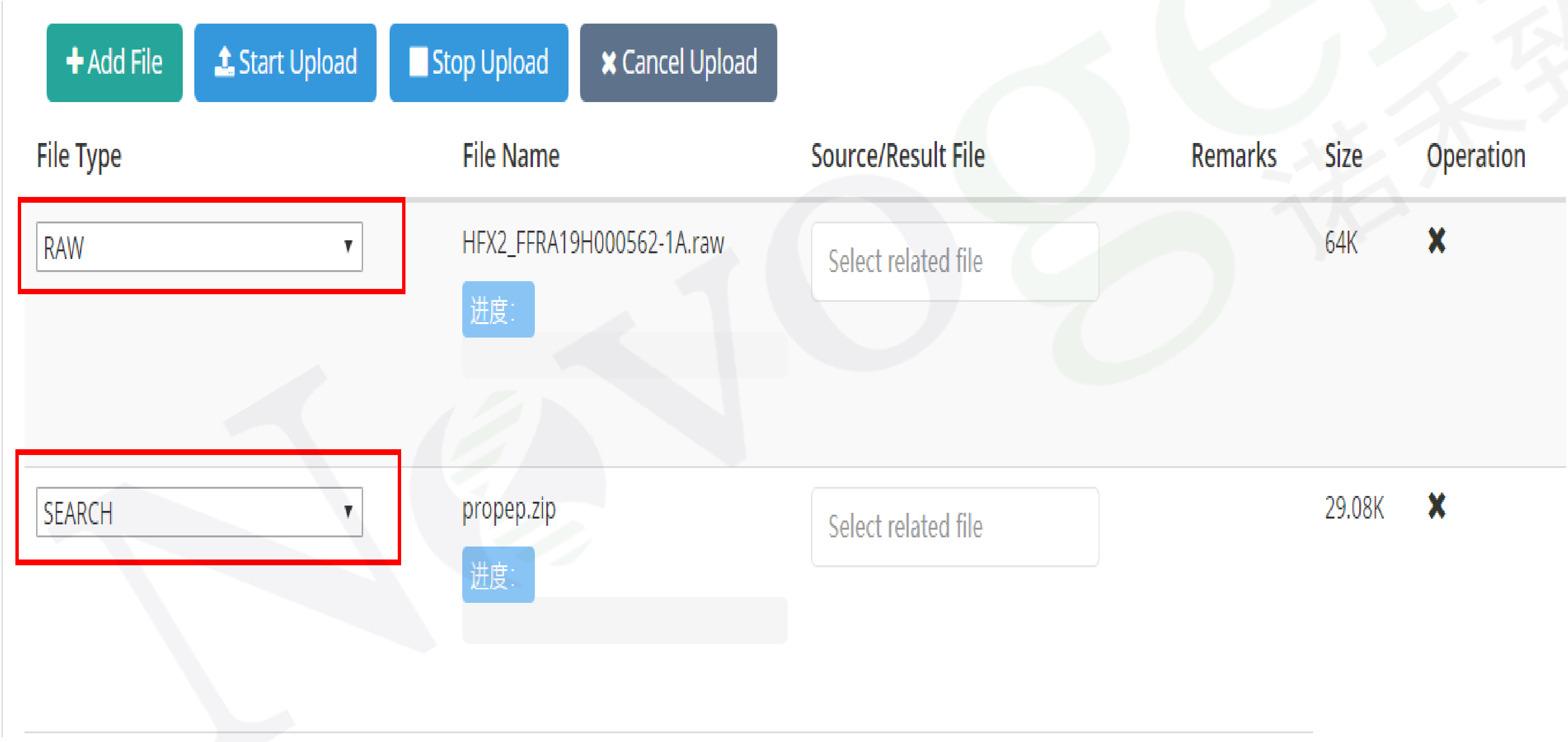

- Complete submission需要同时上传RAW文件和Result文件；
- Partial submission需要同时上传RAW文件和Search文件。
- 添加完之后点击“Start upload”即可开始上传。

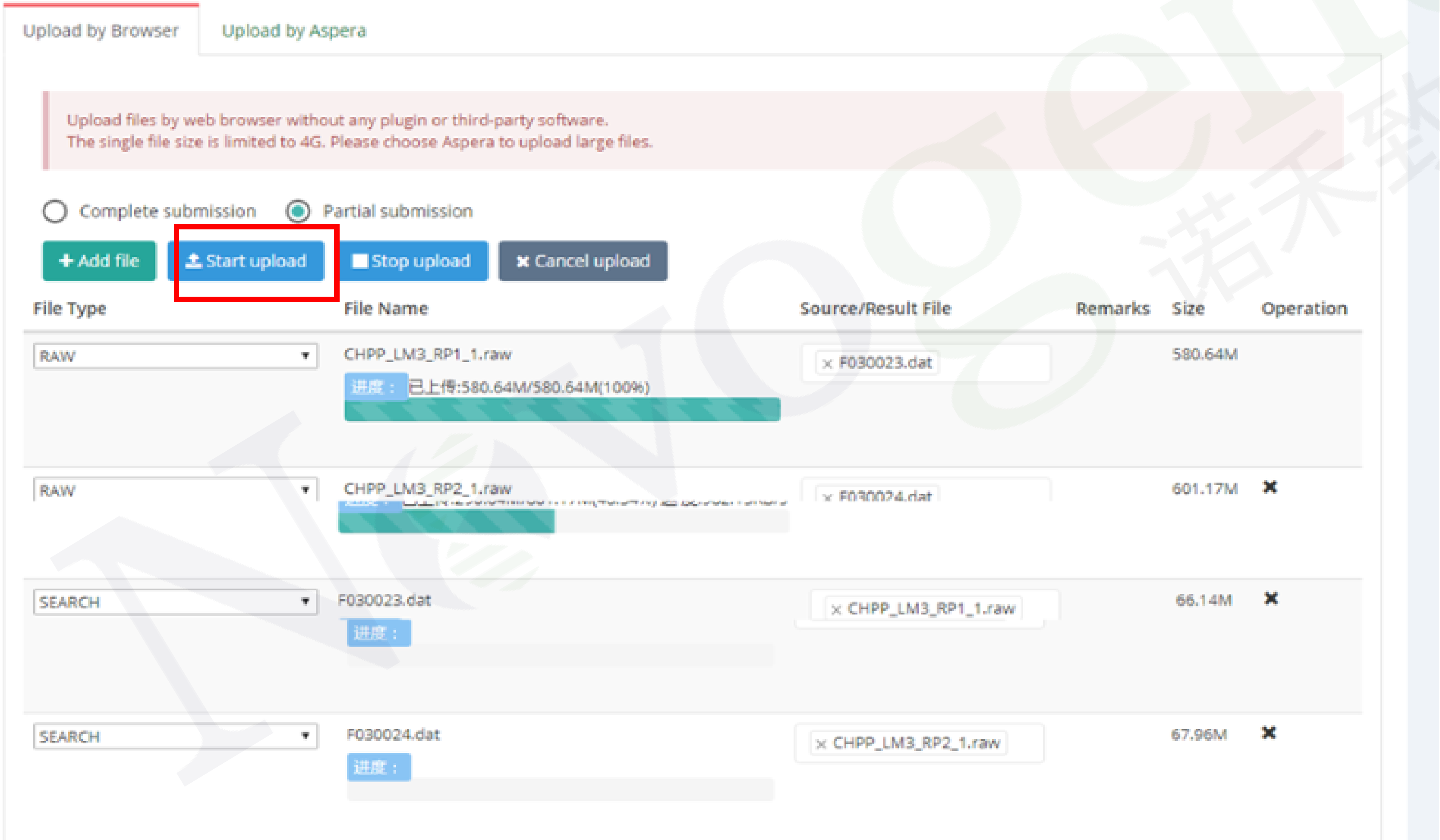

4、Submit

核实上传内容，点击Sumit就完成了整个提交数据的流程。管理者会对上传数据进行审核。

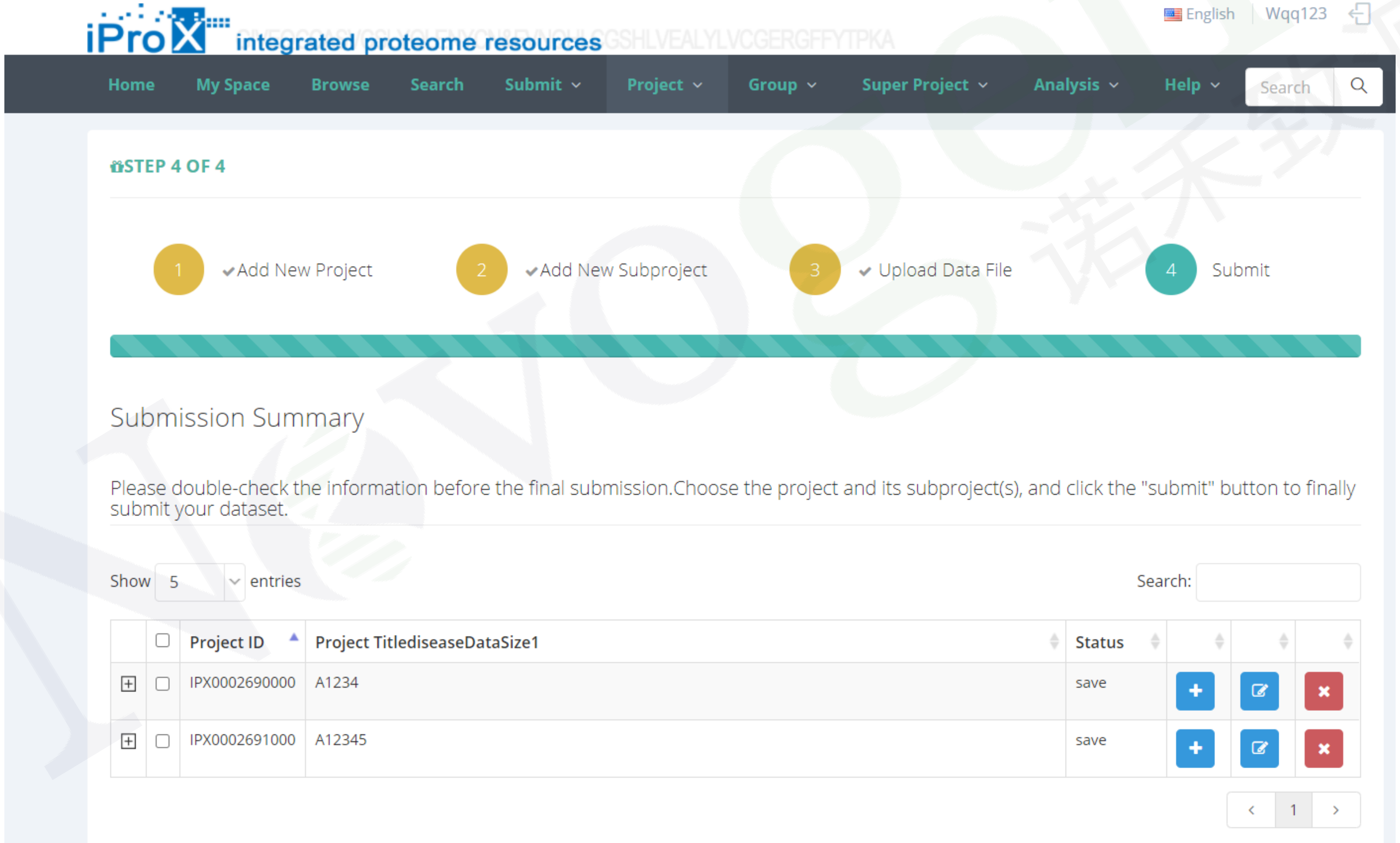

Thank You !

www.novogene.com

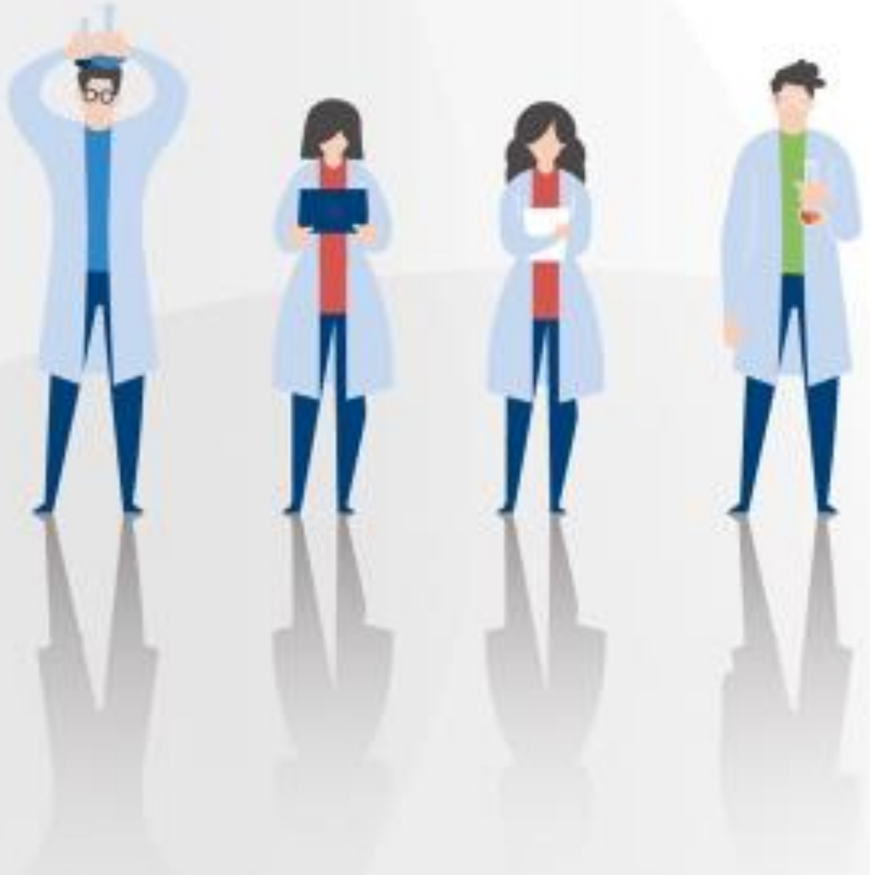

Supplement: Supplementary file 1 — Supplementary Information 1. [file 41598_2022_24687_MOESM1_ESM.zip › raw data/Proteomic raw data/results_readme.pdf]
